# Explaining ethnic disparities in lung function among young adults: A pilot investigation

**DOI:** 10.1371/journal.pone.0178962

**Published:** 2017-06-02

**Authors:** Neil J. Saad, Jaymini Patel, Cosetta Minelli, Peter G. J. Burney

**Affiliations:** National Heart and Lung Institute, Imperial College London, London, United Kingdom; Telethon Institute for Child Health Research, AUSTRALIA

## Abstract

**Background:**

Ethnic disparities in lung function have been linked mainly to anthropometric factors but have not been fully explained. We conducted a cross-sectional pilot study to investigate how best to study ethnic differences in lung function in young adults and evaluate whether these could be explained by birth weight and socio-economic factors.

**Methods:**

We recruited 112 university students of White and South Asian British ethnicity, measured post-bronchodilator lung function, obtained information on respiratory symptoms and socio-economic factors through questionnaires, and acquired birth weight through data linkage. We regressed lung function against ethnicity and candidate predictors defined *a priori* using linear regression, and used penalised regression to examine a wider range of factors. We reviewed the implications of our findings for the feasibility of a larger study.

**Results:**

There was a similar parental socio-economic environment and no difference in birth weight between the two ethnic groups, but the ethnic difference in FVC adjusted for sex, age, height, demi-span, father’s occupation, birth weight, maternal educational attainment and maternal upbringing was 0.81L (95%CI: -1.01 to -0.54L). Difference in body proportions did not explain the ethnic differences although parental immigration was an important predictor of FVC independent of ethnic group. Participants were comfortable with study procedures and we were able to link birth weight data to clinical measurements.

**Conclusion:**

Studies of ethnic disparities in lung function among young adults are feasible. Future studies should recruit a socially more diverse sample and investigate the role of markers of acculturation in explaining such differences.

## Introduction

Low lung volume is strongly related to mortality [1,2]. This is of particular importance for ethnic minorities, for which disparities in lung function are well recognised [3,4]. In the United Kingdom (UK), South Asian British and Black Caribbean/African British, have lower FVC compared to White British individuals across the life course. Differences in FEV_1_/FVC are generally very small and therefore often ignored [5–7]. The ethnic differences in FVC and FEV_1_ persist after adjustment for height, age and sex. Attempts to explain these differences have centred on a difference in chest size, for which proxies such as sitting height or the ratio of sitting to standing height (also termed leg to trunk ratio) are commonly used [5,8]. However, to date, these anthropometric factors have failed to explain the full difference in lung function, suggesting that other factors are at least partially responsible for such difference.

The lower educational and socio-economic status of ethnic minorities compared with White individuals [9,10], contributes to the lower lung function in minority groups [11–13]. Nonetheless, a recent systematic review by Braun et al. on ethnic differences in lung function found that only 6% of studies examined socio-economic status as a potential explanatory factor, while 24% of studies did not provide any explanation for the observed difference [14]. Moreover, birth weight, a marker of intra-uterine development strongly influenced by socio-economic factors, is significantly lower among all ethnic minorities [15–17] and associated with later lung function [18]. Therefore, we hypothesised that a difference in birth weight or socio-economic factors in early life between ethnic groups might explain the difference in lung function.

Here, we describe a pilot investigation on differences in lung function among young adults from two ethnic groups in the UK, White and South Asian British. We assessed the feasibility of performing such a study, with a particular focus on recruitment rate, willingness to take part, potential missingness of information and the feasibility of linkage with birth weight data from the UK birth registry. We also obtained preliminary results on both ethnic differences in lung function and potential explanatory factors that might account for these differences, focusing on birth weight and socio-economic factors.

## Methods

### Participants and data collection

Recruitment and data collection are described in detail in S1 Text. The DELBYA pilot study was a cross-sectional study, conducted between January and July 2015, of students at Imperial College London who were aged 18–23 years, born in the UK, defined themselves as White (i.e. White British, Irish, Gypsy or Irish Traveller or other White) or South Asian British (i.e. Indian, Pakistani, Bangladeshi) ethnicity and had at least three grandparents who were born in the UK and other European countries, or in South Asia (Pakistan, India, Nepal, Bangladesh, Bhutan, Sri Lanka) and East Africa (Kenya, Tanzania, Uganda—where many South Asians had migrated to). Participants self-defined their ethnicity based on the ethnic group questions and categories (Table A in S1 Text) recommended by the Office for National Statistics [19]. Volunteers were excluded if they had any contraindication to undertaking a forced respiratory manoeuvre (Details in S1 Text, Section 1).

All clinical measurements (spirometry and anthropometry) were performed according to standard operating procedures. Long bone length (demispan and ulna) were measured twice each [20] using a measuring tape (Seca) and height (0.1 cm accuracy) and weight (0.1 kg accuracy) were measured once, using a stadiometer (Leicester) and weighing scales (Marsden), respectively, with participants asked to remove shoes, items from their pockets and heavy clothing. Spirometery was performed according to ATS/ERS guidelines [21] both pre- and post-bronchodilator. Participants were seated and performed spirometry, using the ndd EasyOne Spirometer (ndd Medizintechnik AG, Zurich, Switzerland), while wearing a noseclip. Participants performed at least three acceptable blows but could attempt up to eight blows. Participants were given two 100-microgram puffs of salbutamol (Salamol, IVAX pharmaceuticals UK) via a valved spacer (ABLE spacer, Clement Clarke International, England) and, following both inhalations, waited at least 15 minutes before performing the post-bronchodilator spirometry. The calibration of the spirometer was checked daily using a 3L syringe and the spirograms were reviewed by a physician (PB), who was blinded to birth weight and ethnicity. A quality score based on the ATS/ERS acceptability and reproducibility criteria was assigned and the highest of the acceptable post-bronchodilator FVC and FEV_1_ values were used in the statistical analyses.

Questionnaires were employed to elicit information on respiratory symptoms, smoking, parental and grandparental socio-economic status and educational attainment, parental and grandparental upbringing and place of birth, as well as feedback on the study methods and procedures (S2 Text). Birth weight was obtained from the Health and Social Care Information Centre (HSCIC), a UK government agency, through linkage of participant’s name, date of birth and NHS number. All study visits took place in the respiratory biomedical research unit at the Royal Brompton Hospital, London, UK.

### Statistical analyses

Data were summarised using mean and standard deviation for continuous variables or number and percentage for categorical variables, and the proportion of missing data was reported. Differences between the two ethnic groups and differences between those who did and did not have birth weight data were investigated using a t-test or, when necessary, the non-parametric Wilcoxon rank sum test for continuous variables, and, due to the low number of participants, Fisher’s exact test for categorical variables. Estimates of the association of risk factors with respiratory symptoms by ethnicity were expressed in terms of odds ratios.

Associations with the main outcome of interest, post-bronchodilator FVC, and FEV_1_/FVC were assessed using linear regression. Differences between the two ethnic groups in the outcomes were assessed using multiple linear regression with the covariates sex, age, height, demispan, father’s occupation, birth weight, maternal educational attainment and maternal upbringing specified a priori, on the basis of the existing literature and the experience of the research group. The variance inflation factor was employed to evaluate multicollinearity [22]. As secondary outcomes, we investigated differences in the risk of respiratory symptoms between the two ethnic groups, expressed as odds ratio.

In an additional analysis, we assessed the association of the outcomes of interest with variables beyond those selected a priori (Table E in S1 Text). Because standard linear regression can result in overfitting when the sample size is small relative to the number of variables tested in the model, we employed penalised regression. Penalised regression methods address the issue of overfitting by shrinking the regression coefficients towards zero [23]. We chose lasso (least absolute shrinkage and selection operator) regression since it simultaneously estimates the parameters and performs variable selection by shrinking some of the coefficients exactly to zero and therefore removing them from the model [23]. The lasso was used for further exploratory analyses investigating a larger number of potential predictors, since this technique addresses the problem of model overfitting. More detail on the lasso regression is available in S1 Text.

All analyses were conducted in Stata 13.1 (StataCorp LP, College Station, TX, USA), apart from the lasso regression, which was performed using the glmnet package [24] in R 3.2.4 (R Foundation for Statistical Computing, Vienna, Austria). Statistical significance was defined as p<0.05.

### Ethical approval

Ethical approval for the study was obtained from the Imperial College Research Ethics Committee (ICREC 13_6_16) and the Imperial College Medical Education Ethics Committee (MEEC 1314–07). Written informed consent was obtained from each participant.

## Results

### Study conduct and participants’ feedback

From a target population of 3,863 UK students at Imperial College London, aged 18–23, and of White or South Asian British ethnicity 112 (3%) participants were recruited into the study, with no difference in recruitment by ethnicity (S1 Fig). All 112 participants who completed the study visit responded to all questions and participated in all procedures or measurements, but missing information occurred when participants did not know the answer to a question, which was predominantly the case for questions related to the grandparents’ upbringing, educational attainment and occupational status (Table F in S1 Text).

Twenty-six participants provided feedback for the study, of which 23 had attended the study visit (20.5% of those who attended the study visit) and 3 had not (13% of those who provided contact details but did not attend the study visit). The feedback questionnaire showed that all participants reported that they were “very comfortable” with all study procedures, apart from seven reporting to be only “somewhat comfortable” with spirometry (S3 Fig). No adverse effects from spirometry were observed during the study visit. All participants agreed that the information sheet provided sufficient information and those who did not attend the study visit cited lack of time as a reason. The study visit did not take place at the Imperial College London main campus but only one participant found travelling to the Royal Brompton Hospital difficult. A third of participants (n = 7) found that the study visit lasted longer than expected and four participants reported that the reimbursement was too little. These participants suggested a reimbursement ranging from £20 to £30.

Reasons for taking part were equally divided between helping medical research, being interested in knowing their lung function and for the reimbursement (S4 Fig). Recruitment flyers appeared the most successful for the recruitment of study participants, while the other methods, through friends, shout-outs during lectures, clubs and societies or social media, performed markedly worse (S5 Fig).

### Study population

**Fig 1:**
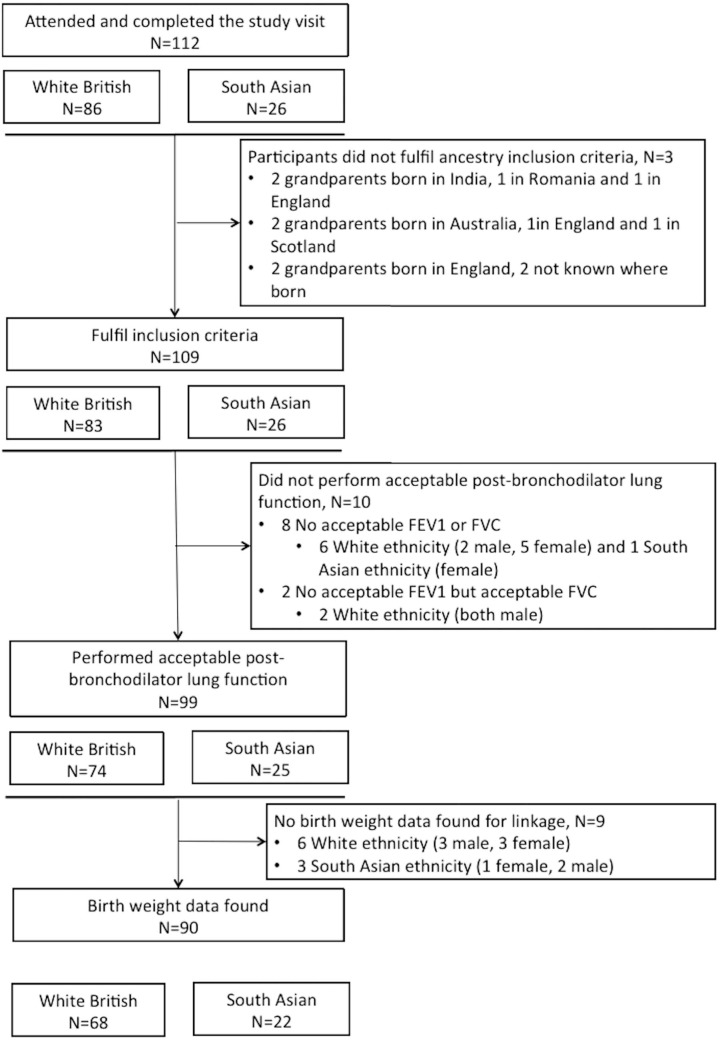
Flow chart of participants.

Of the 112 participants recruited into the study, three White participants that did not fulfil the ancestry inclusion criterion were excluded, while poor lung function test quality excluded a further 10 participants (Fig 1). Birth weight could be obtained for 90 of the participants with acceptable lung function, which resulted in a sample size of 68 White and 22 South Asian British participants (Fig 1). Those without birth weight data were slightly older than those with (21.2 vs. 20.4 yrs; p = 0.050) but did not differ in FVC, FEV_1_ or FEV_1_/FVC.

The characteristics of the participants are shown in Table 1. Half of the study population were women, equally divided among White and South Asian British students. White participants had higher birth weight and were younger, taller and heavier, but had lower BMI and shorter demi-span and ulna length compared to South Asian British participants, although none of these differences were statistically significant. Only seven participants (8%), had ever smoked cigarettes and only one, a South Asian British participant, had ever smoked a waterpipe. Similarly, exposure to environmental tobacco smoke did not differ between the two ethnic groups nor were the differences in proportions of smoking of either parents or of a parent, mother or father, significant (Table 1, Table G in S1 Text).

**Table 1:** Characteristics of the DELBYA study population.

	White (N = 68)	South Asian British (N = 22)	
	Mean (SD) or N (%)	Mean (SD) or N (%)	P-value
Sex (Female)	35 (51.5)	12 (54.2)	0.80
Age (yrs)	20.3 (1.2)	20.9 (1.5)	0.08^b^
**Anthropometry**			
Height (cm)	173.7 (8.3)	169.5 (12.5)	0.07
Weight (kg)	67.3 (10.3)	67.0 (11.3)	0.92^b^
BMI (kg/m^2^)	22.2 (2.7)	23.3 (2.9)	0.12
Demispan (cm)	80.6 (4.4)	80.9 (6.0)	0.92^b^
Ulna (cm)	26.2 (1.8)	26.7 (2.5)	0.73^b^
**Smoking**			
Ever smoked, cigarettes	6 (8.8)	1 (4.5)	1.00
Ever smoked, pack-years (n)	0.5 (0.5)	2.5 (0.0)	-
Passive smoke exposure, people smoke where you live	11 (16.2)	3 (13.6)	1.00
Passive tobacco smoke exposure, at home	4 (5.9)	1 (4.6)	1.00
Passive tobacco smoke exposure, in social settings	12 (17.7)	6 (27.3)	0.36
Passive tobacco smoke exposure, elsewhere	7 (10.3)	3 (13.6)	0.70
Parental smoking, before pregnancy	22 (32.4)	5 (22.7)	0.44
Parental smoking, during pregnancy	8 (11.8)	0 (0)	0.19
Parental smoking, during childhood	9 (13.2)	3 (13.6)	1.00
Parental smoking, nowadays	6 (8.8)	2 (9.1)	1.00
**Early life influence**			
Birth weight (kg)	3.4 (0.5)	3.3 (0.6)	0.76
**Parental immigration status**			
Both parents born in the United Kingdom	54 (79.4)	1 (4.6)	<0.001
One parent born in the United Kingdom	13 (19.1)	3 (13.6)	
No parent born in the United Kingdom	1 (1.5)	18 (81.8)	
**(Grand)Parental upbringing**^a^ (urban/rural)			
Mother, rural	16 (23.5)	8 (36.4)	0.27
Maternal grandmother, rural	21 (35.6)	14 (73.7)	0.007
Maternal grandfather, rural	21 (40.4)	13 (68.4)	0.059
Father, rural	13 (19.1)	9 (40.9)	0.049
Paternal grandmother, rural	19 (33.9)	15 (75.0)	0.003
Paternal grandfather, rural	14 (26.9)	16 (80.0)	<0.001
**(Grand)Parental educational attainment**^a^			
Mother (≤High School)	15 (22.1)	4 (18.2)	1.00
Maternal grandmother (≤High School)	40 (71.4)	11 (73.3)	1.00
Maternal grandfather (≤High School)	31 (58.5)	9 (52.9)	0.78
Father (≤High School)	14 (20.6)	4 (18.2)	1.00
Paternal grandmother (≤High School)	36 (67.9)	12 (85.7)	0.32
Paternal grandfather (≤High School)	31 (62.0)	11 (64.7)	1.00

^a^Response to questions for (grand)parental upbringing and educational attainment was incomplete for some participants with details on the missing data are provided in S1 Text.

^b^P values derived using a Wilcoxon rank-sum test.

Most South Asian British participants (n = 18, 82%) were second-generation immigrants (neither parent born in the UK), whereas for the majority of White participants both parents were born in the UK (n = 54, 79%). Parents and grandparents of South Asian British participants were also more likely to have had a rural upbringing compared to White participants. All South Asian British participants had three or more grandparents born in South-Asia, except one participant whose grandparents were born in either South-Asia or East-Africa. Conversely 88% of White British participants had at least three grandparents who were born in the UK (n = 60), while the remaining participants had at least three grandparents who were born in either the UK or Western Europe (n = 6) or were born solely in Western Europe (n = 2).

Despite the difference in upbringing and immigration status, the educational attainment of parents or grandparents did not differ between South Asian British and White participants. The occupational status of the parents and grandparents were very similar and no difference was observed in the father’s or mother’s occupation nor in the paternal or maternal grandfathers’ jobs. However, grandmothers of South Asian British participants were much more likely to have never worked outside the home (Tables H and I in S1 Text).

### Lung function and respiratory symptoms

Wheezing was markedly different between White and South Asian British participants (Table 2). South Asian British participants showed a higher prevalence of wheezing or whistling, odds ratio (OR) of 3.6 (95% CI: 1.2 to 10.4), wheezing in the absence of a cold (OR: 3.7; 95% CI: 1.0 to 14.3) and more serious wheezing that made participants feel short of breath (OR: 6.4; 95% CI: 1.4 to 29.4). Despite the difference in wheezing over the last 12 months, no difference in the prevalence of asthma diagnosed by a physician was observed (16% in White and 23% in South Asian British). Few participants reported any other respiratory symptoms (exacerbation in the last year, chronic cough, being hospitalised as a child, breathlessness) and these did not differ significantly between the ethnic groups (Table 2).

**Table 2:** Lung function and respiratory symptoms by ethnicity.

		White British (N = 68)	South Asian British (N = 22)	Effect estimate^a^ (95% CI) (ref: White British)	P-value
		Mean (SD) or N (%)	Mean (SD) or N (%)		
**Lung function**					
FVC (L)		4.7 (0.9)	3.8 (0.9)	-0.86 (-1.32;-0.40)	<0.001
FVC (% Predicted^b^)		97.1 (10.3)	94.8 (10.4)	-2.32 (-7.36;2.72)	0.36
FEV_1_ (L)		4.1 (0.8)	3.4 (0.9)	-0.71 (-1.11;-0.32)	<0.001
FEV_1_ (% Predicted^b^)		99.6 (10.7)	97.2 (9.9)	-2.37 (-7.49;2.75)	0.36
FEV_1_/FVC (%)		87.6 (5.4)	88.1 (6.3)	0.56 (-2.18;3.29)	0.69
**Respiratory symptoms**^c^					
Chronic cough		1 (1.5)	1 (4.5)	3.2 (0.2;53.2)	0.42
Asthma, ever		11 (16.2)	5 (22.7)	1.5 (0.5;5.0)	0.49
Asthma, current		4 (5.9)	2 (9.1)	1.6 (0.3;9.4)	0.60
Wheezing, last year		11 (16.2)	9 (40.9)	3.6 (1.2;10.4)	0.02
Wheezing, last year not only when a cold		5 (7.4)	5 (22.7)	3.7 (1.0;14.3)	0.06
Wheezing, last year made you feel short of breath		3 (4.4)	5 (22.7)	6.4 (1.4;29.4)	0.02
Any exacerbation, ever		8 (11.1)	1 (4.5)	0.4 (0.04;3.0)	0.35
Any exacerbation, last year		2 (2.9)	0 (0.0)	-	-
Dyspnea (MRC scale)				3.2 (0.2;53.2)	0.42
1		67 (98.5)	21 (95.5)		
≥1		1 (1.5)	1 (4.0)		
Hospitalised as child for breathing problems		4 (5.9)	2 (9.1)	1.6 (0.3;9.4)	0.60

^a^ Effect estimate refers to differences in lung function or odds ratio for respiratory symptoms

^b^ % Predicted obtained using NHANES III reference equations with a correction factor of 0.88 for South Asian British participants

^**c**^A description of the respiratory symptoms is provided in Table B in S1 Text

We did not include ulna length as a confounder as it was too strongly correlated with the demi-span. When adjusted for all potential confounders selected *a priori*, the difference in FVC between ethnic groups fell slightly from 0.86 L (95% CI: -1.32 to -0.40 L) to 0.81 L (95% CI: -1.01 to -0.54 L) (Table 3). Maternal education beyond high school was positively associated with FVC in the adjusted model; however, fathers in professional occupations had children with on average 0.27 L (95% CI: 0.0069 to 0.53L) lower FVC (Table 3).

**Table 3:** Crude and adjusted difference in FVC and FEV1/FVC across categories (categorical variables) and per unit (continuous variables).

	FVC (L)				FEV1/FVC (%)			
	Crude		Adjusted^a^		Crude		Adjusted^a^	
	Difference (95% CI)	P-value	Difference (95% CI)	P-value	Difference (95% CI)	P-value	Difference (95% CI)	P-value
**Ethnicity** (South Asian British)	-0.86 (-1.32;-0.40)	<0.001	-0.81 (-1.01;-0.54)	<0.001	0.56 (-2.2;3.3)	0.69	0.38 (-3.07;3.84	0.83
**Sex** (Female)	-1.51 (-1.81;-1.27)	<0.001	-0.90 (-1.23;-0.56)	<0.001	0.30 (-2.05;2.66)	0.80	-1.00 (-5.27;3.28)	0.64
**Age** (yrs)	-0.10 (-0.26;0.05)	0.19	0.047 (-0.029;0.12)	0.22	0.053 (-0.83;0.94)	0.91	-0.035 (-1.02;0.95)	0.94
**Height** (cm)	0.09 (0.07;0.10)	<0.001	0.025 (-0.005;0.055)	0.10	-0.067 (-0.19;0.057)	0.29	-0.064 (-0.45;0.32)	0.74
**Demispan** (cm)	0.15 (0.12;0.18)	<0.001	-0.036 (-0.016;0.089)	0.17	-0.12 (-0.37;0.12)	0.33	-0.068 (-0.75;0.61)	0.84
**Father’s occupation** (≥professional category)	0.027 (-0.53; 0.58)	0.92	-0.27 (-0.53;-0.0069)	0.044	1.02 (-2.05;4.09)	0.51	0.92 (-2.45;4.29)	0.54
**Birth weight** (kg)	0.29 (-0.10;0.68)	0.14	0.052 (-0.15;0.26)	0.61	-1.19 (-3.35;0.98)	0.28	-0.78 (-3.40;1.84)	0.56
**Maternal educational attainment** (>High school)	0.58 (0.076;1.09)	0.025	0.33 (0.064;0.59)	0.015	-0.031 (-2.91;2.85)	0.98	0.22 (-3.19;3.62)	0.90
**Maternal upbringing** (Urban)	0.12 (-0.35;0.60)	0.60	-0.077 (-0.31;0.15)	0.50	-0.11 (-2.77;2.55)	0.93	-0.0075 (-2.95;2.93)	0.99

^a^Difference for each predictor adjusted for ethnicity, sex, age, height, demispan, father’s occupation, birth weight, maternal educational attainment and maternal upbringing.

**Fig 2:**
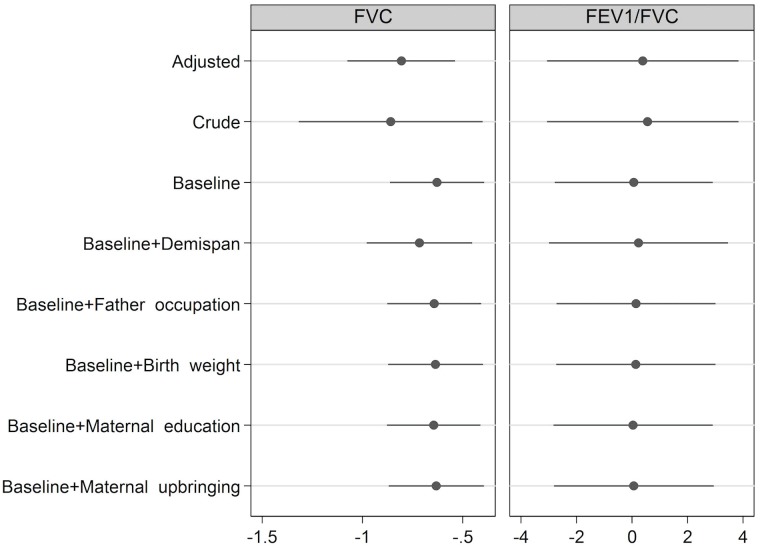
Crude and adjusted difference in L (FVC) or % (FEV1/FVC) of South Asian British vs White participants. “Baseline” refers to adjustment for height and sex. The adjusted model includes adult height, sex, demispan, father’s occupation, birth weight, maternal educational attainment and maternal upbringing.

Fig 2 shows the impact of the adjustment for individual covariates on the estimate of the association of ethnicity on FVC. None of the socio-economic factors (father’s occupational status, maternal educational attainment or maternal upbringing) nor birth weight were able to explain the difference in FVC. Adjusting for demi-span did not reduce the difference in FVC between ethnic groups, but rather increased it by 15%, although this was not statistically significant (Fig 2). FEV_1_/FVC did not show any statistically significant association with ethnicity, sex, age, birth weight or any reported anthropometric or parental measures (Table 3, Fig 2), while FEV_1_ showed similar results to FVC (Table J in S1 Text).

**Fig 3:**
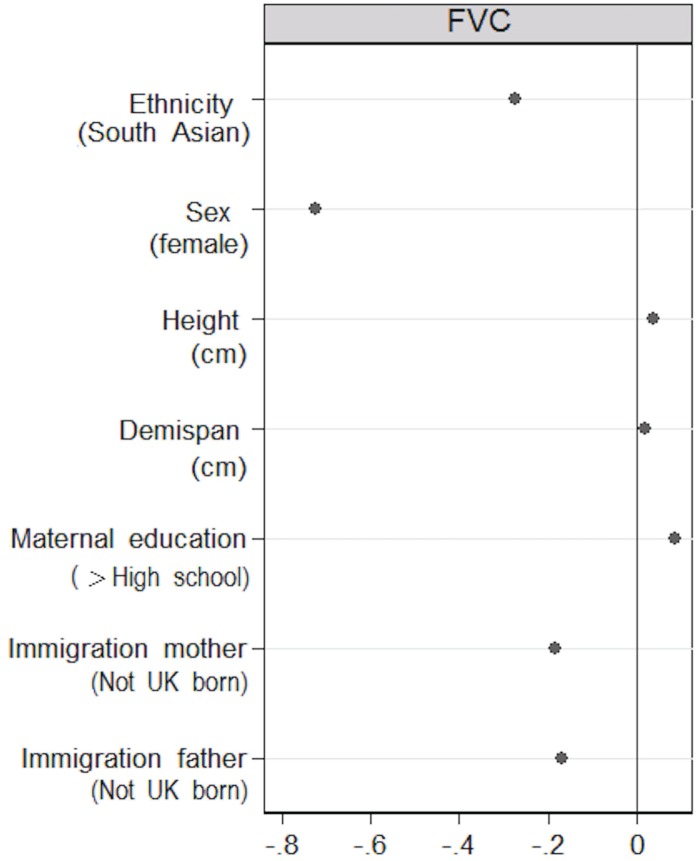
Adjusted difference in L for FVC for predictors identified by the lasso regression.

The additional analysis with lasso regression identified six predictors of FVC, in addition to ethnicity (Fig 3). These included sex, height, demi-span and maternal educational attainment, in agreement with the results from the linear regression in the main analysis, although the effect estimates were attenuated, as expected, due to the shrinkage of the lasso regression. Two additional predictors, not included in the main analysis, were identified by the lasso regression, the immigration status of the mother and the father, which were independently associated with lower FVC. The effect of the parents’ combined immigration status on FVC was more pronounced than that of the participant’s own self-defined ethnicity. As in the main analysis, the lasso regression did not identify ethnicity as a predictor of FEV_1_/FVC (data not shown).

## Discussion

This pilot investigation was able to recruit a young adult sample and link their birth weight data to clinical measurements. The study found a difference in FVC despite the similar socio-economic status of the parents and the lack of any difference in birth weight between the two ethnic groups. Thus, it is unlikely that birth weight or socio-economic factors can fully explain the difference in FVC between the two ethnic groups in this study population. Moreover, this pilot investigation found no support for the hypothesis that a difference in body proportions can explain ethnic differences in lung function and identified parental immigration as an important predictor of FVC.

### Interpreting study findings

This pilot study showed an FVC 0.86 L lower in South Asian compared with White participants, while no difference was observed for FEV_1_/FVC. These findings are in line with two previous studies, performed in different age groups. In the BOLD London study in British adults older than 40, Hooper et al. found the FVC 0.79 L and 0.49 L lower in South Asian British compared with Whites, for men and women respectively [7]. Similarly, Strippoli et al. observed an FVC 0.31 L lower in 9 to 14 year old South Asian British compared with White children, after adjusting for age, sex, weight and height [6]. No difference in FEV_1_/FVC between White and South Asian British subjects was observed in the BOLD London study [7], while a higher FEV_1_/FVC was reported by Strippoli et al. [6] in South Asian British children.

In our study, South Asian British students were 3.6 times more likely to experience wheezing in the last year compared to White students, although we observed no significant difference in doctor-diagnosed asthma between the two ethnic groups or in reversibility of lung function following the administration of bronchodilator (200 micrograms salbutamol). This could be a result of differences in reporting symptoms, due to differences in the perception and experience of wheezing or cultural differences in reporting symptoms. Alternatively, there could be a true difference in environmental and genetic risk factors between the two groups that results in more wheezing among South Asian British students independent of lung function reversibility.

Parental and grandparental factors were very similar in the two ethnic groups. No differences in educational attainment of parents and grandparents or in occupational status of parents or grandfathers between the two ethnic groups were observed, though South Asian paternal and grandparental upbringing was more likely to have occurred in a rural environment and South Asian grandmothers were more likely to have been housewives. Although it was rare for both parents of South Asian British participants to be born in the UK, they were professionally and educationally very similar to the parents of the white British students. The study participants from both ethnic groups appear to have grown up in similar socio-economic but different cultural environments. This is in contrast with the low parental socio-economic environment of South Asian British compared to White children in the study by Strippoli et al. [6]. The similar socio-economic environment in this pilot study reflects patterns of recruitment at an elite university and future studies should expand the recruitment to cover the entire width of the social class distribution.

We did not observe a difference in birth weight between the two ethnic groups while Strippoli et al. [6] found a significantly lower birth weight, of 226 grams, in South Asian British vs White children. The comparable socio-economic environment may explain the lack of difference in birth weight between the two ethnic groups. A recent meta-analysis provided strong evidence for an association of birth weight with FVC in adulthood [18] with an estimated increase of 59.4mL per kilogram increase in birth weight. This is close to our estimate in this study of 52 mL per kilogram increase in birth weight (95CI%: -15 to 26), and the lack of statistical significance is likely to be due to the small sample size.

Long-bone length relative to other aspects of body size, commonly assessed as either the ratio of arm span to height or the ratio of sitting to standing height, has been associated with social advantage and lower mortality [25–28]. However, relatively longer long bones will reduce the comparative length of the trunk (and hence of the thorax) relative to standing height. African Americans compared to Whites have relatively longer long bones [3,29], and therefore differences in lung function between ethnic groups when adjusted for standing height have been attributed to the reduced thoracic size relative to standing height. For this reason sitting height has been preferred as a predictor of lung function when comparing different ethnic groups [5,30,31]. However, sitting height is difficult to measure accurately, and in this pilot study we assessed two other measures that include a measurement of long bone length; ulna length and demi-span, which measures both long bone length (humerus, radius and ulna) and chest width. Both ulna length and demi-span were easily measured although, due to multicollinearity, measuring only one would suffice in future studies. Adjusting for long-bone length did not reduce the estimated difference in FVC between the ethnic groups; it increased by 15% although this was not statistically significant. The direction of this change is contrary of what would be expected if long-bone length explained the lower height–adjusted FVC in ethnic minorities, as has been suggested [5,30,31]. If greater long bone length were responsible for the lower height-adjusted lung volumes in South Asian students, adjustment would have reduced, not increased, the difference between the groups. Although differences in body proportions did not explain ethnic differences in lung function in our study, this could be further examined in future studies, possibly using more detailed chest and lung function measurements, such as chest circumference, sitting height or plethysmography. The difference in FVC between the White and South Asian British students was substantial in spite of the very similar birth weights and socio-economic background of the participants’ parents and grandparents. Moreover, the difference in FVC in this pilot study was comparable to that observed in a different study population of adults (the BOLD London study [7]). Therefore, it is unlikely that the difference in birth weight or parental socio-economic variables might fully explain the difference in FVC between the ethnic groups not only in this study population but likely in other populations as well.

The immigration status of the mother and father were identified by the lasso regression as important predictors of FVC, with their combined effect being larger than the effect estimate for (self-defined) ethnicity. This suggests an environmental influence explaining the difference in lung function between ethnic groups, which is potentially related to cultural factors that would be continuing to have an effect post-migration. However, findings from our lasso analysis should be regarded as preliminary and require further confirmation in future studies. The investigation of factors that might affect the offspring’s lung function and might be associated with parental migration also requires information on the timing of the migration, as this could affect the exposure and duration of exposure.

Potential environmental factors to investigate in future studies include exposure to culturally specific sources of air pollution, such as incense burning or shisha smoking in the home, or other toxins imported from the country of origin, as occurred with the importation of cosmetics such as Kohl, a product that contained high levels of heavy metals [32–34]. Diet also is an important cultural factor that differs by ethnic group and would persist following migration. Carey et al. [35] showed that differences in the risk of bronchial hyperactivity and atopy among White and Asian British school children could be explained by the extent to which children had adopted a Western diet. There is increasing evidence supporting the influence of nutrition on later lung function. A randomized clinical trial among pregnant mothers in Nepal found that offspring of pregnant mothers who were given vitamin A supplementation had significantly higher FVC and FEV_1_ at age 9 to 13 years compared to children in the placebo group [36]. Devakumar failed to reproduce this result but their study used lower doses of Vitamin A in a trial of multi-vitamin supplements [37]. Devereux and coworkers [38] showed in observational studies that reduced maternal vitamin E and zinc during pregnancy was associated with a higher risk of wheeze and asthma while reduced α-tocopherol was associated with lower FEV_1_. Subsequently, they reported that a low maternal level of α-tocopherol level was associated with a reduced FVC, possibly mediated by a reduced crown-rump length at the first trimester scan [39]. These studies provide plausibility that differences between ethnic groups in maternal or childhood diets might be able to explain some of the differences in early adult lung function. Future studies could investigate this using food frequency questionnaires or using dietary biomarkers [40–42].

Although exposures continuing after migration are the most likely explanation we cannot exclude epigenetic factors with persistent effects in the offspring born outside the country of origin of the parents, or other exposures during extended visits back to the original family home during childhood, and these need to be recorded carefully in future studies [43–45]. Moreover, even though it is unlikely that socio-economic factors can fully explain the difference in FVC between the two ethnic groups, socio-economic status is a complex construct, associated with occupation, education and income, and it is possible that we may not have been able to capture the socio-economic environment fully, despite the collection of detailed educational and occupational information in this pilot investigation. Therefore, future studies should further explore a possible role for different aspects of socio-economic status.

### Evaluation of pilot study design and recommendations for future studies

#### Participant recruitment

Previous studies investigating the difference in lung function between White and South Asian British participants found marked differences in socio-economic status [5,6]. However, the recruitment at an elite university in the pilot investigation was likely responsible for the comparable socio-economic environment in both ethnic groups. Although future studies should focus on environmental influences related to parental immigration, these studies should ensure that the entire width of the social class distribution is covered. The selection of a socio-economically diverse study population will allow for the investigation of environmental influences across social classes and enables, as a secondary aim, the investigation of socio-economic factors as explanatory factor. Expanding the study population can be achieved by recruiting from a more diverse range of academic institutions, which could range from elite universities to technical colleges, and among vocational training programs in companies or manual occupations, to include those that enter the workforce directly from school.

Participation into epidemiological studies has severely declined in the past decades [46] and only 3% of participants from the target population were recruited into the study (S1 Fig), similar to the recruitment rate of a recent cohort study in the UK, the UK COSMOS study [47]. Although 92% of the target study population did not attempt to take part (S1 Fig), the estimated size of the target population has probably been overestimated, being inflated by the presence of ineligible students. These could not be always be identified from the College records as we have no information on the country of birth of students or their grandparents. Therefore, the recruitment percentage should be considered as a lower bound estimate. Nonetheless, steps could be taken to improve recruitment in future studies by identifying potential barriers. The largest gain in this pilot investigation could have been made by increasing participation in the screening. Performing the study on-site, including the initial screening questionnaires using tablets or other mobile devices, could lower the barrier for participation by increasing the presence and visibility of the study and eliminating travel time. All of those who did not take part in the study but provided feedback on their non-participation, reported lack of time as the reason for non-participation, which suggests that extending the times available for study visits, for instance after classes in the evening or for those working during the day, could have increased participation rates. Finally, to increase retention of participants questionnaires should be kept as succinct as possible. For example, the fact that 56 students who were eligible did not complete the online registration questionnaire, suggests that this questionnaire should be shortened.

#### Data collection and linkage

Compliance with the study procedures and the completeness of information for the questionnaires were very good. Only a small number of participants were unable to perform acceptable post-bronchodilator lung function after coaching due to poor coordination of in- and exhalation, while other missing data were observed only for questions pertaining to grandparental information. This might be explained by reduced contact with grandparents due to geographic distance or early death.

Birth weight linkage was possible for nearly all participants and was provided by NHS Digital from the ONS birth dataset, which is considered the most complete dataset for birth weight information [48]. However, it took almost eight months to acquire the birth weight data. Alternative datasets exist but contain markedly more missing data and are both also managed by NHS Digital [48]. An alternative approach could be to obtain birth weight data from GP practices. However, in the Size and Lung function in Children study this resulted in heightened non-response (33% of GPs did not respond to data requests) and birth weight information was only obtained for 47% of participants. Moreover, GPs non-response or presence of missing birth weight information was higher for those of non-White British ethnicity and those with lower family socio-economic status [49]. Finally, participants were not asked for self-reported birth weight. Although this was not a problem in our study, which focused on youngsters born in the UK for whom birth weight data are available, future studies may consider also collecting self-reported birth weight to enable the inclusion of participants born outside the UK and of older participants for which birth registry data are not available. However, self-reported birth weight is influenced by recall bias, with women more likely to recall their birth weight than men [50]. Additionally, self-reported birth weight is prone to measurement error; a study in women showed that only 28% could report their birth weight within 4 ounces of the birth weight recorded in the registry while 47% did not know their birth weight and 28% reported it inaccurately (>4 ounces difference from their registry birth weight) [50]. Therefore, the reliability of self-reported birth weight for young adults would need to be ascertained prior to the inclusion of these data.

## Conclusion

Disparities in lung function between ethnic groups have long been recognised, but findings to date have not provided conclusive answers on what might explain them. This pilot investigation showed that it is feasible to study this question among University students and provides the information required to plan a more definitive study. It has identified parental immigration as a potential predictor of FVC, and found no evidence that differences in birth weight or body proportions explained the ethnic differences in lung function. Future studies should widen the social background of participants and focus on markers of acculturation, such as diet, to explain the ethnic differences.

## Supporting information

S1 Fig Flow chart of the recruitment into the study. (PNG)

S2 Fig Flow chart of study visit and procedures. (PNG)

S3 Fig Comfort of procedures during study visit. (PNG)

S4 Fig Motivation for participation in the study. Some participants indicated multiple reasons for participation and therefore the numbers do not add up to 26.
(PNG)

S5 Fig Means of recruitment. (PNG)

S1 Text Detailed study methods and additional results. (DOCX)

S2 Text Study questionnaires. (PDF)
