# Supplementary figures and images for: Explaining ethnic disparities in lung function among young adults: A pilot investigation
Source: PLoS One. 2017 Jun 2;12(6):e0178962. doi: 10.1371/journal.pone.0178962 (PMC5456386; doi:10.1371/journal.pone.0178962)

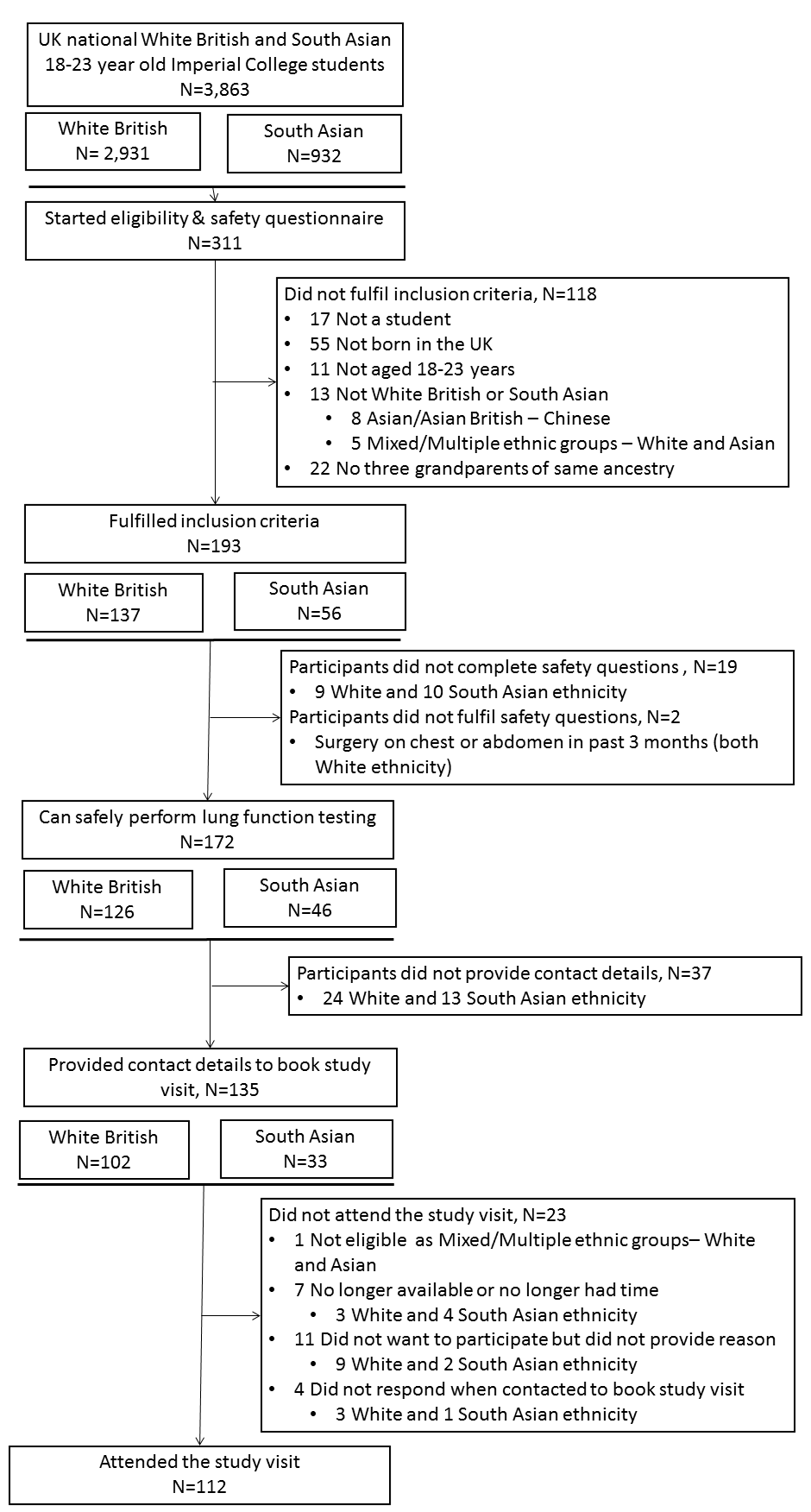

Supplement: S1 Fig — (PNG) [file pone.0178962.s001.png]

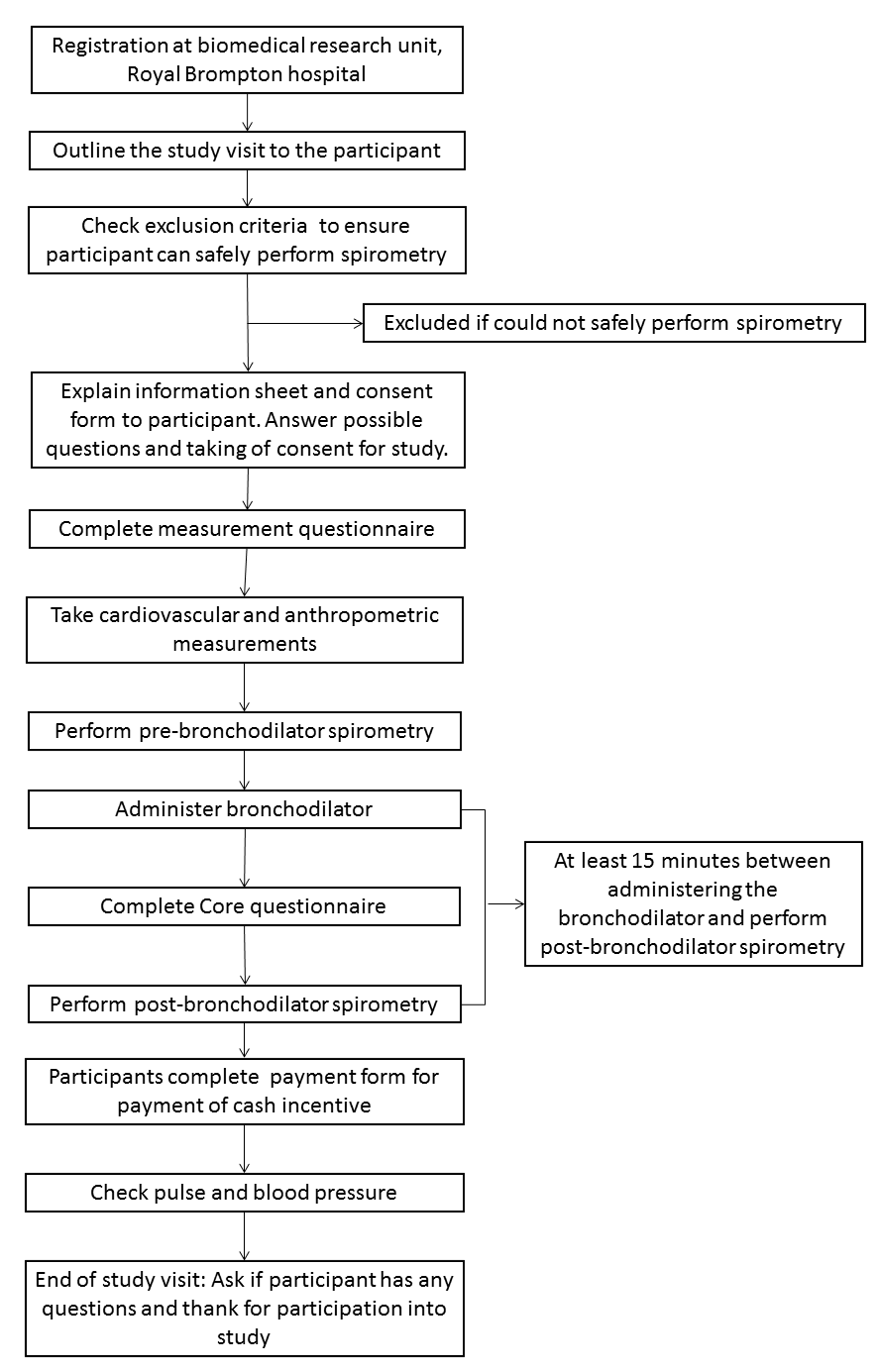

Supplement: S2 Fig — (PNG) [file pone.0178962.s002.png]

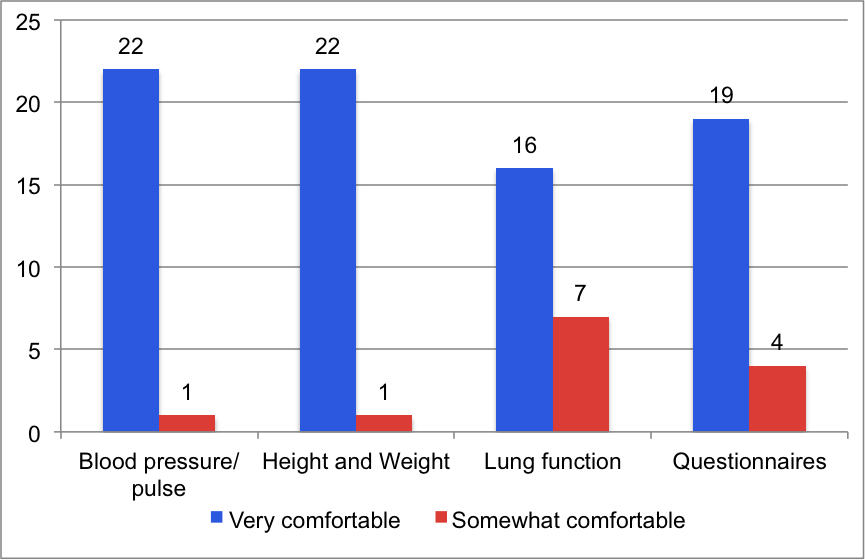

Supplement: S3 Fig — (PNG) [file pone.0178962.s003.png]

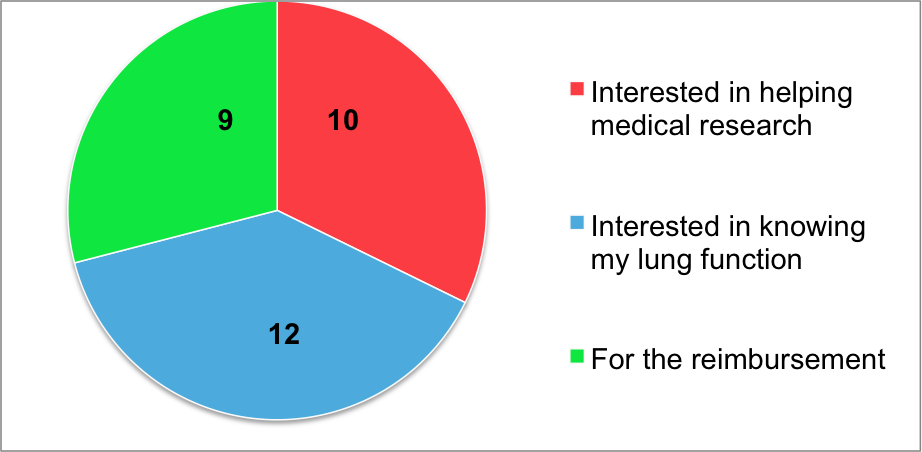

Supplement: S4 Fig — Some participants indicated multiple reasons for participation and therefore the numbers do not add up to 26. (PNG) [file pone.0178962.s004.png]

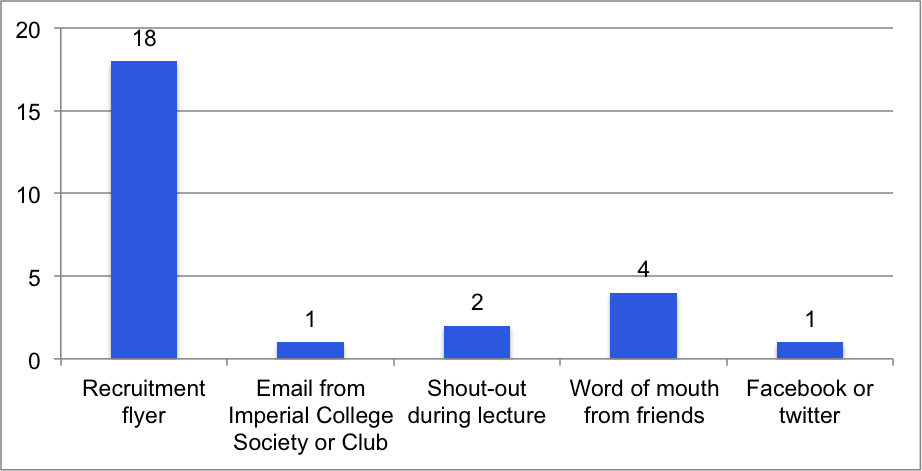

Supplement: S5 Fig — (PNG) [file pone.0178962.s005.png]
