# Supplementary material for: Explaining ethnic disparities in lung function among young adults: A pilot investigation
Source: PLoS One. 2017 Jun 2;12(6):e0178962. doi: 10.1371/journal.pone.0178962 (PMC5456386; doi:10.1371/journal.pone.0178962)
Supplement: S1 Text — (DOCX) [file pone.0178962.s006.docx]

**S1 Text**

**Explaining ethnic disparities in lung function among young adults: a pilot investigation**

Neil J. Saad^1^, Jaymini Patel^1^, Cosetta Minelli^1^, Peter Burney^1^

^1^ National Heart and Lung Institute, Imperial College London, UK

1. **Participant recruitment**

Participants were recruited from Imperial College London and offered a cash incentive of £15 for participation in the study, which was based on the UK minimum hourly wage and the total time the participants spent travelling and undergoing the clinical examination.

Participants were recruited through advertisements on noticeboards in College buildings, and on online College noticeboards, advertisements in Imperial College Union Club & Society newsletters, shout-outs after lectures following the approval of the lecturer, and advertisements on social media (Imperial College Facebook and Twitter)

Participants were excluded if they had any of the following contraindications to undertaking a forced respiratory manoeuvre:

1. Surgery on the chest or abdomen in the last three months
2. Heart attack in the past three months
3. Having a detached retina or having had eye surgery in the past three months
4. Been hospitalised for any other heart problem in the past month
5. Being in the last trimester of pregnancy
6. Currently taking medication for tuberculosis
7. Having had a pneumothorax in the last three months

Figure S1 provides an overview of the recruitment, with details on dropouts and exclusions. Over 3,800 students at Imperial College London were a UK national, which was considered as a proxy for being born in the UK, of White or South Asian British ethnicity (Table S1) and aged between 18 and 23 during the recruitment period. Of the 311 individuals who started the online registration questionnaire, 172 individuals were eligible for inclusion in the study and could safely perform spirometry. The main reason for not fulfilling the inclusion criteria was not being born in the UK (n=55, 47% of all excluded), while two students were excluded for recent surgery on chest or abdomen. Contact details were obtained for 135 students who were invited to the study visit of whom 112 attended. Thus overall 112 (3%) participants were recruited into the study from a target population of 3,863 UK students at Imperial College London, aged 18-23, and of White or South Asian British ethnicity during the period of the study. There was no difference in recruitment by ethnicity: 86 White participants took part (2.9%, 2,931 in the target population) vs 26 South Asian British students (2.8%, 932 in the target population).

1. **Study data and visit**
   1. **Study questionnaires**

Study data were recorded on five questionnaires:

- 1. Registration questionnaire prior to the study visit: Assessed the inclusion/exclusion criteria and obtained participants’ contact details to book the study visit.
  2. Safety questionnaire during the study visit: Reassessed the exclusion criteria at the time of the study visit.
  3. Measurement questionnaire during the study visit: Recorded the results from the anthropometric measurements and the compliance of, or adverse events to, spirometry.
  4. Core questionnaire during the study visit: Recorded information on respiratory symptoms, smoking, parental and grandparental socio-economic status and educational attainment, and parental and grandparental upbringing and place of birth, which was based on the Burden of Obstructive Lung Disease (BOLD) study core questionnaire [1]
  5. Feedback questionnaire after the study visit: Collected feedback on the study conduct and procedures.
  6. **Questionnaire for respiratory symptoms and risk factors**

Information on chronic cough, asthma, wheezing, exacerbations, dyspnea and hospitalisation for breathing problems were collected, with an overview and description provided in Table S2. Active smoking and exposure to environmental tobacco smoke (including parental smoking) throughout the life course were assessed, with Table S3 showing the definitions used in the study. Parental and grandparental socio-economic and ancestry information is shown in Table S4. An International Standard Classification of Occupations (ISCO) code was allocated, using the ISCO-88 classification [2], based on the description of the usual job or occupation, type of work and industry. In the statistical analyses the major groups of the ISCO-88 classification were used to group the parents and grandparents.

- 1. **Demispan and ulna measurements**

Demispan was measured standing, with the right arm extended horizontally from the shoulder, from between the middle and ring finger of the right hand to the mid-point of the sternal notch. The ulna was measured on the left arm, while the participant was seated and with the participant bending the arm across the chest with the palm flat and fingers pointing toward the right shoulder, from the point of the elbow (olecranon) to the mid-point of the prominent bone of the wrist (ulna styloid process) [3].

- 1. **Study visit**

Figure S2 provides an overview of the procedures conducted during the visit. After the initial registration, assessment of contraindications for a forced expiratory manoeuvre (exclusion criteria) and study consent, participants’ cardiovascular and anthropometric measurements were taken, as was pre-bronchodilator lung function. The core questionnaire was completed after the administration of the bronchodilator, which was followed by the measurement of the post-bronchodilator lung function. The study visit was concluded by checking the participants’ blood pressure and pulse rate before they left the hospital.

1. **Statistical analyses: lasso regression**

In an additional analysis, we wanted to assess the association of the outcomes of interest (post-bronchodilator FVC and FEV_1_/FVC) with all variables for which information was available. However, standard linear regression results in overfitting when the sample size is small relative to the number of variables tested in the model. We therefore employed penalised regression, where the problem of overfitting is handled by placing a constraint on the regression coefficients and shrinking them towards zero [4]. In particular, we chose the lasso (least absolute shrinkage and selection operator) regression, which performs variable selection in addition to parameter estimation, thus providing a reduced final model with the set of variables that maximises the predictive ability of the model [5]. The number of variables kept in the final model depends on the value of the parameter controlling the amount of shrinkage, which is chosen based on cross-validation [6]. All variables tested in the lasso regression for both FVC and FEV_1_/FVC are listed in Table S5.

1. **Study population: additional findings**
   1. **Missing data**

Although all participants who completed the study visit responded to all questions and participated in all procedures or measurements, missing information occurred when participants did not know the answer to a question or, in the case of lung function, because the measurement did not meet the quality standard. No post-bronchodilator FVC or FEV_1_ was available for ten participants (9.2% of those who fulfilled the inclusion criteria. Table S6 provides an overview of the other missing information in the study. There was no missing information for any of the questions related to respiratory symptoms or smoking history. All participants but one were aware of their parents’ smoking history, but up to a quarter of participants could not recall information on their grandparents’ upbringing, educational attainment or occupational status.

- 1. **Exposure to parental smoking**

The proportions of parents, either the mother or father, smoking across the life course of the participant did not vary between the two ethnic groups (Table S7).

- 1. **Occupational status of parents and grandparents**

No difference was observed in the occupation of parents’ or paternal grandparents’ between the two ethnic groups. However, grandmothers of South Asian British participants were more likely to never have worked (Table S8 and S9).

1. **Lung function and respiratory symptoms: additional findings**
   1. **Association analyses for FEV_1_**

The results from the association analyses for FEV_1_ were comparable to the results for FVC. Table A.10 shows the crude and adjusted differences for FEV_1_. FEV_1_was also lower among women, while both anthropometric measures (height and demi-span) and birth weight showed a positive relation with FEV_1_, with all of these associations being statistically significant except for birth weight. As all participants were aged between 18 and 23 years, age did not influence FEV_1_. Paternal and maternal educational and socio-economic variables were also positively associated with FEV_1_, but this was only statistically significant for maternal educational attainment. In the adjusted analyses, the difference in FEV_1_ between the ethnic groups was slightly reduced from 0.71 L (95% CI: -1.10 to -0.32 L) to 0.66 L (95% CI: -0.89 to -0.42 L). The effect estimates of sex on FEV_1_ was reduced while the association of demi-span and height with FEV_1_ became not statistically significant. Maternal educational attainment remained positively associated with FEV_1_ in the adjusted model but, in contrast, father’s occupational status and maternal upbringing negatively affected FEV_1_.

- 1. **Lung function reversibility**

Lung function reversibility was assessed by comparing the difference of pre- to post-bronchodilator lung function relative to the post-bronchodilator lung function [12]. As expected, the post-bronchodilator was statistically significantly higher than pre-bronchodilator lung function but only one participant varied by more than 12.5% in FEV_1_ (Table S11).

- 1. **Family history of respiratory disease and adverse influences for spirometry**

No participant reported a family history of COPD, emphysema or chronic bronchitis. Post-bronchodilator FVC, FEV_1_ or FEV_1_/FVC were also not influenced by a cold in the last three weeks, by whether participants had smoked in the last month, or by any medications for breathing in the last 24 hours before performing spirometry (p>0.05, Wilcoxon rank-sum test).

**References**

1 Buist a S, Vollmer WM, Sullivan SD, *et al.* The Burden of Obstructive Lung Disease Initiative (BOLD): rationale and design. *Copd* 2005;**2**:277–83. doi:10.1081/COPD-200057610

2 International Labour Organisation. International Standard Classification of Occupations. 1988.

3 Todorovic V, Russell C, Stratton R, *et al.* The malnutrition universal screening tool explanatory booklet. 2003.

4 Pavlou M, Ambler G, Seaman SR, *et al.* How to develop a more accurate risk prediction model when there are few events. *British Medical Journal* 2015;**351**:h3868. doi:10.1136/bmj.h3868

5 Tibshirani R. Shrinkage and Selection via the Lasso. *Journal of the Royal Statistical Society Series B (Methodological)* 1996;**58**:267–88. doi:10.2307/2346101

6 Friedman J, Hastie T, Tibshirani R. Regularization Paths for Generalized Linear Models via Coordinate Descent. *Journal of Statistical Software* 2010;**33**:1--22.

7 Toledano MB, Smith RB, Brook JP, *et al.* How to establish and follow up a large prospective cohort study in the 21st century - Lessons from UK COSMOS. *PloS one* 2015;**10**:e0131521. doi:10.1371/journal.pone.0131521

8 Ghosh RE, Ashworth DC, Hansell AL, *et al.* Routinely collected English birth data sets: comparisons and recommendations for reproductive epidemiology. *Archives of disease in childhood Fetal and neonatal edition* 2016;**101**:F451-7. doi:10.1136/archdischild-2015-309540

9 Bonner R, Bountziouka V, Stocks J, *et al.* Birth data accessibility via primary care health records to classify health status in a multi-ethnic population of children: an observational study. *NPJ primary care respiratory medicine* 2015;**25**:14112. doi:10.1038/npjpcrm.2014.112

10 Lawlor D, Davey Smith G, Ebrahim S. Birth weight is inversely associated with coronary heart disease in post-menopausal women: findings from the British women’s heart and health study. *Journal of epidemiology and community health* 2004;**58**:120–5.

11 Hollis RB, Was CA. Mind wandering, control failures, and social media distractions in online learning. *Learning and Instruction* 2016;**42**:104–12. doi:10.1016/j.learninstruc.2016.01.007

12 Tan WC, Vollmer WM, Lamprecht B, *et al.* Worldwide patterns of bronchodilator responsiveness: results from the Burden of Obstructive Lung Disease study. *Thorax* 2012;**67**:718–26. doi:10.1136/thoraxjnl-2011-201445

13 Fletcher CM, Elmes PC, Fairbairn a S, *et al.* The significance of respiratory symptoms and the diagnosis of chronic bronchitis in a working population. *British medical journal* 1959;**2**:257–66. doi:10.1136/bmj.2.5147.257

**Figure legends**

**Figure S1:** Flow chart of the recruitment into the study

**Figure S2:** Flow chart of study visit and procedures

**Figure S3:** Comfort of procedures during study visit

**Figure S4:** Motivation for participation in the study

**Figure S5:** Means of recruitment

**Figure S1**


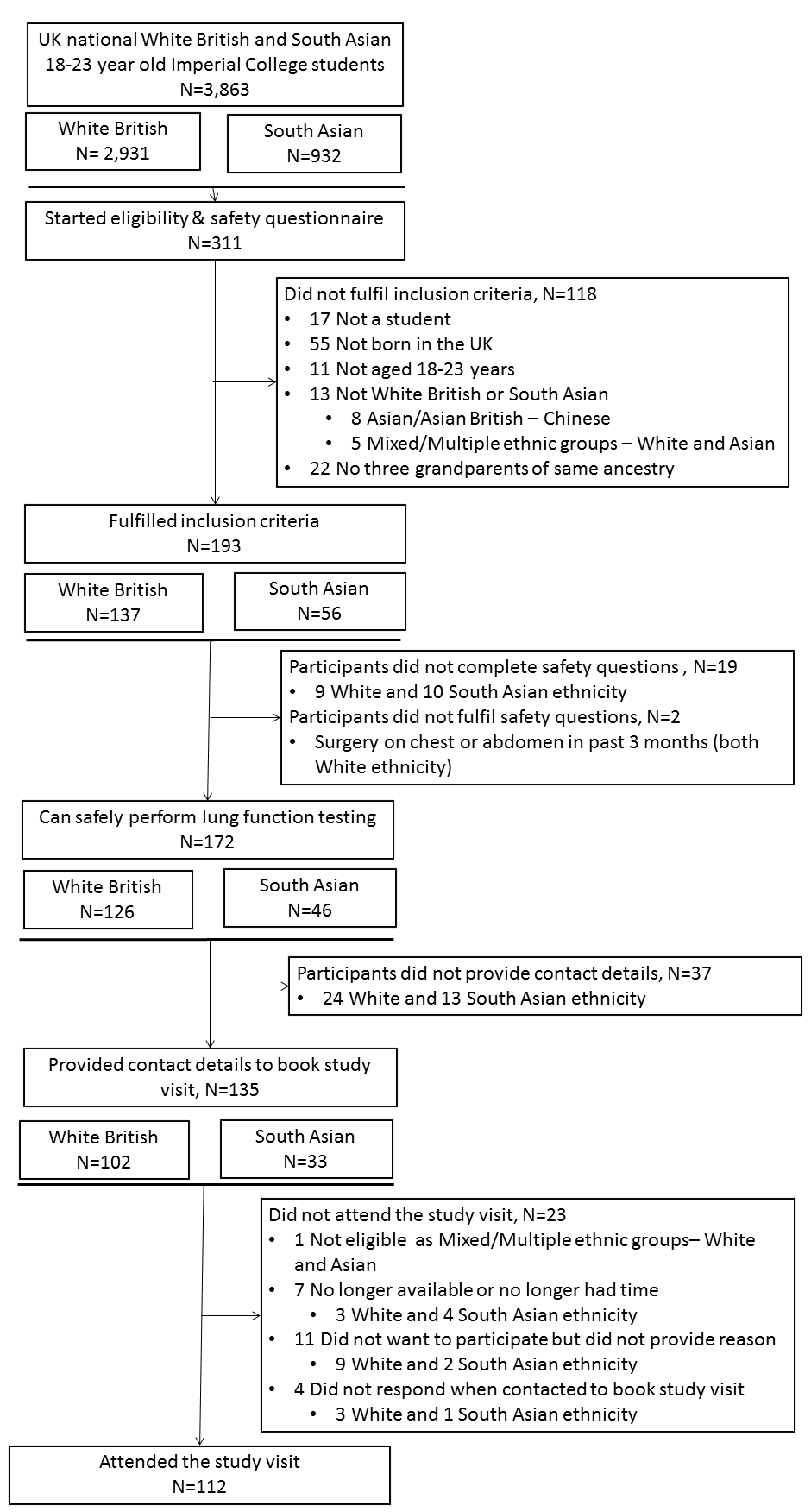


**Figure S2**


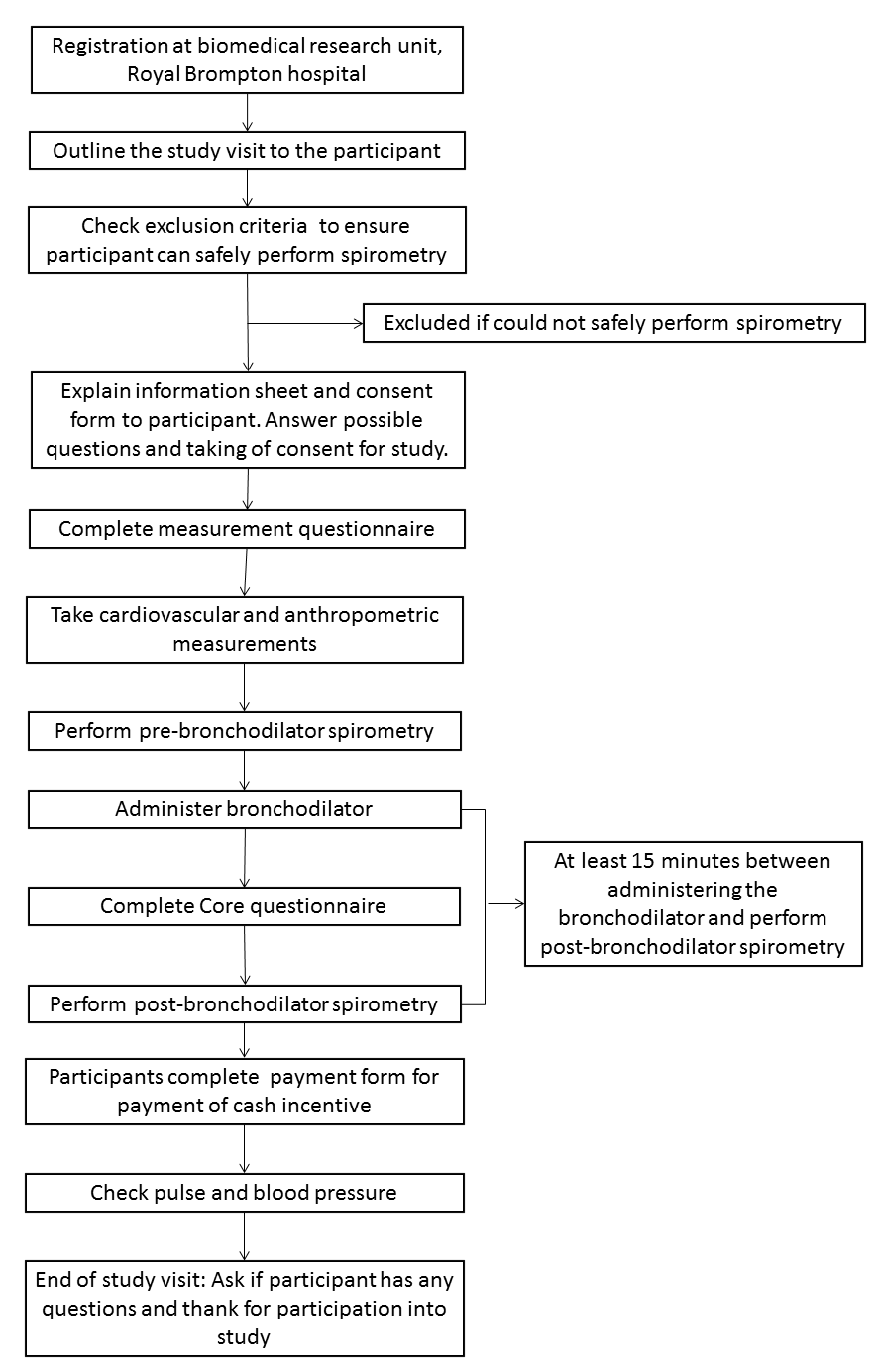


**Figure S3**


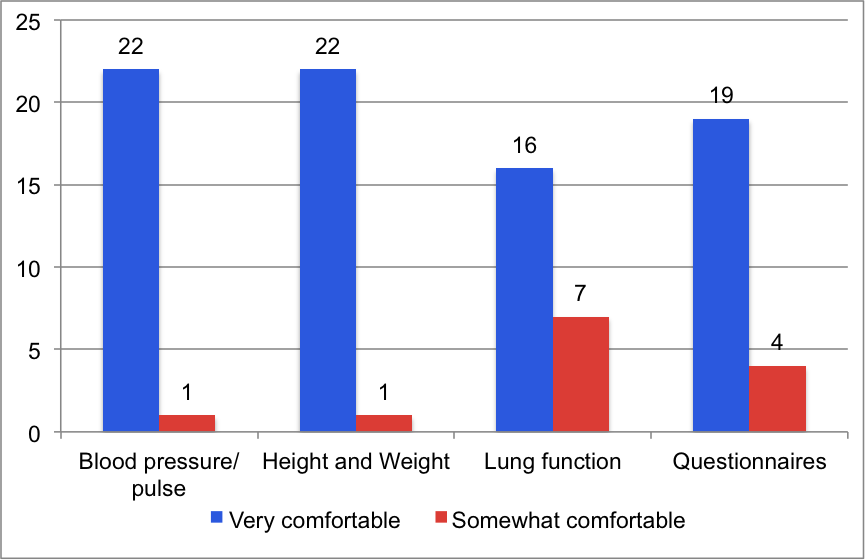


**Figure S4**


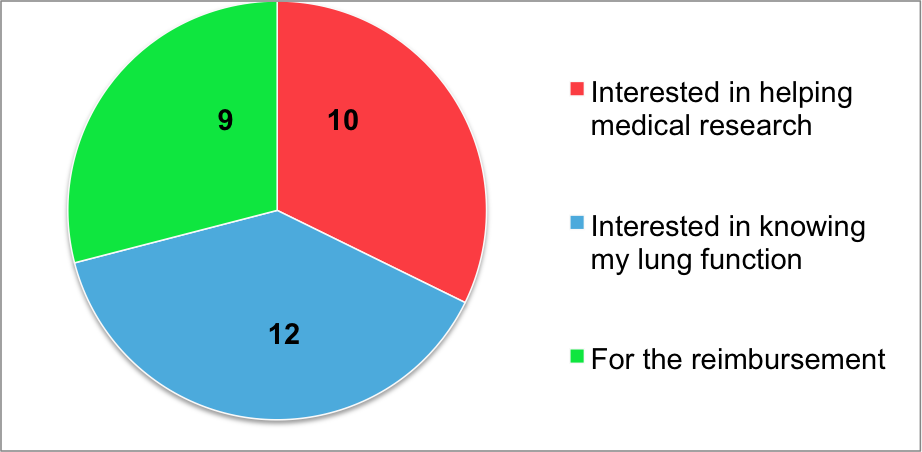


Note: Some participants indicated multiple reasons for participation and therefore the numbers do not add up to 26

**Figure S5**


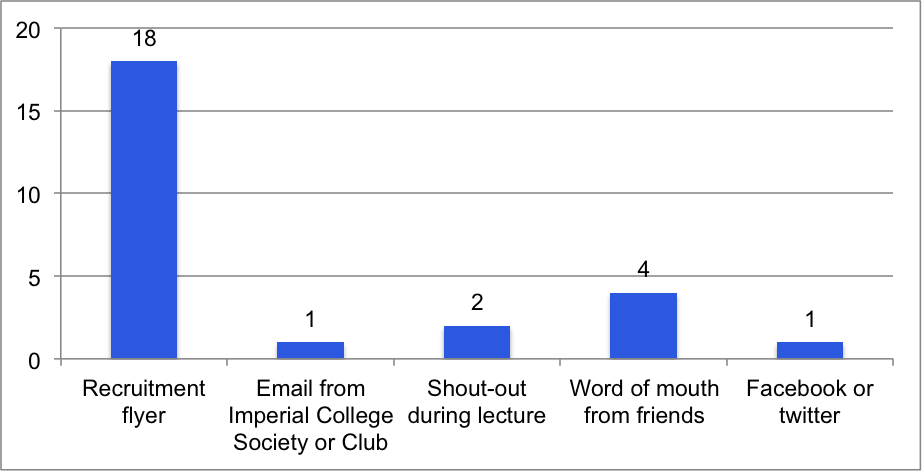


**Table S1: Ethnic group question from the Office for National Statistics**

| **White** |  |
| --- | --- |
| English / Welsh / Scottish / Northern Irish / British | ❑ |
| Irish | ❑ |
| Gypsy or Irish Traveller | ❑ |
| Any other White background, please describe:  __________________ | ❑ |
|  |  |
| **Mixed / Multiple ethnic groups** |  |
| White and Black Caribbean | ❑ |
| White and Black African | ❑ |
| White and Asian | ❑ |
| Any other Mixed / Multiple ethnic background, please describe:  ___________________ | ❑ |
|  |  |
| **Asian / Asian British** |  |
| Indian | ❑ |
| Pakistani | ❑ |
| Bangladeshi | ❑ |
| Chinese | ❑ |
| Any other Asian background, please describe:  ___________________ | ❑ |
|  |  |
| **Black / African / Caribbean / Black British** |  |
| Caribbean | ❑ |
| African | ❑ |
| Any other Black / African / Caribbean background, please describe:  ___________________ | ❑ |
|  |  |
| **Other ethnic group** |  |
| Arab | ❑ |
| Any other ethnic group, please describe:  __________________ | ❑ |

**Table S2:** Description of respiratory symptoms

| **Variable** | **Categories** | **Note** |
| --- | --- | --- |
| Chronic cough | No/Yes | Affirmative answer to all subsequent questions:   1. ‘Do you usually cough when you don’t have a cold’? 2. ‘Are there months in which you cough on most days?’ 3. ‘Do you cough on most days for as much as three months each year?’   And confirmation that coughs lasts at least 2 years in a single choice question: ‘For how many years have you had this cough?’ |
| Asthma, ever | No/Yes | Answer to “Has a doctor or other health care provides ever told you that you have asthma, asthmatic bronchitis or allergic bronchitis?” |
| Asthma, current | No/Yes | Answer to “Do you still have asthma, asthmatic bronchitis or allergic bronchitis?” |
| Wheezing, last year | No/Yes | Answer to “Have you had wheezing or whistling in your chest at any time in the last 12 months?” |
| Wheezing, last year not only when a cold | No/Yes | Answer to “Have you had this wheezing or whistling only when you have a cold?” |
| Wheezing, last year made you feel short of breath | No/Yes | Answer to “In the last 12 months, have you ever had an attack of wheezing or whistling that has made you feel short of breath?” |
| Any exacerbation, ever | No/Yes | Answer to “Have you ever had a period when you had breathing problems that got so bad that they interfered with your usual daily activities or caused you to miss work?” |
| Any exacerbation, last year | No/Yes | Derived from the question “How many such episodes have you had in the past 12 months” which relates to any exacerbation was “Yes” was defined as having any exacerbation in the past year. |
| Dyspnea | 1-5 | Five point modified medical research council scale [13] |
| Hospitalised as child for breathing problems | No/Yes | Answer to “Where you hospitalized as a child for breathing problems prior to the age of 10?” |

**Table S3:** Description of smoking variables

| **Variable** | **Categories** | **Note** |
| --- | --- | --- |
| Ever smoked cigarettes | No/Yes | Answer to “Have you ever smoked cigarettes?” with “Yes” meaning more than 20 packs of cigarettes in a lifetime or more than 1 cigarette each day for a year. |
| Pack-years |  | Derived from the number of years smoked and number of packs of cigarettes smoked per year |
| Passive smoke exposure, people smoke where you live | No/Yes | Derived from the question “Not counting yourself, how many people in your household smoke regularly?”. “Yes” means at least one person smokes in the household. |
| Passive smoke exposure, at home | No/Yes | Derived from the question “How many hours per day are you exposed to other people’s tobacco smoke at home?”. “No” means not exposed (i.e. 0 hours/day). |
| Passive smoke exposure, in social settings | No/Yes | Derived from the question “How many hours per day are you exposed to other people’s tobacco smoke in bars, restaurants, cinemas or similar social settings?”. “No” means not exposed (i.e. 0 hours/day). |
| Passive smoke exposure, elsewhere | No/Yes | Derived from the question “How many hours per day are you exposed to other people’s tobacco smoke elsewhere?”. “No” means not exposed (i.e. 0 hours/day). |
| Parental smoking, before pregnancy | No/Yes | Derived from the questions “Did your mother smoke regularly before your pregnancy?” and “Did your father smoke regularly before your pregnancy?”. “Yes” means at least one parent smoked. |
| Parental smoking, during pregnancy | No/Yes | Derived from the questions “Did your mother smoke regularly during your pregnancy?” and “Did your father smoke regularly during your pregnancy?”. “Yes” means at least one parent smoked. |
| Parental smoking, during childhood | No/Yes | Derived from the questions “Did your mother smoke regularly during your childhood?” and “Did your father smoke regularly during your childhood?”. “Yes” means at least one parent smoked. |
| Parental smoking, nowadays | No/Yes | Derived from the questions “Does your mother smoke regularly nowadays?” and “Does your father smoke regularly nowadays?”. “Yes” means at least one parent smoked. |

**Table S4:** Overview of immigration, upbringing and ancestry variables in the DELBYA study

| **Variable** | **Categories** | **Note** |
| --- | --- | --- |
| **Immigration** |  |  |
| Immigration status | a) Both parents born in the UK; b) One parent born in the UK; c) No parent born in the UK | Derived from the place of birth of the parents |
| **Upbringing** |  |  |
| Mother | a) Rural; b) Urban | Derived from the question “What term best describes the place your parent/grandparent lived most of the time during her childhood?”. “Rural” is defined as farm or village in a rural area. “Urban” is defined as Small town or suburb or city. |
| Maternal grandmother | a) Rural; b) Urban |  |
| Maternal grandfather | a) Rural; b) Urban |  |
| Father | a) Rural; b) Urban |  |
| Paternal grandmother | a) Rural; b) Urban |  |
| Paternal grandfather | a) Rural; b) Urban |  |
| **Ancestry** |  |  |
| Grandparental ancestry |  | Derived from the place of birth of the grandparents. |
| **Educational attainment** |  |  |
| Mother | a) ≤High School; b) >High School | Derived from the question “What is the highest level of schooling your parent/grandparent has completed?” |
| Maternal grandmother | a) ≤High School; b) >High School |  |
| Maternal grandfather | a) ≤High School; b) >High School |  |
| Father | a) ≤High School; b) >High School |  |
| Paternal grandmother | a) ≤High School; b) >High School |  |
| Paternal grandfather | a) ≤High School; b) >High School |  |
| **Occupation** |  |  |
| (Grand)parental occupation | Classified in “Did not work” or ISCO-88 major groups: a) Armed forces; b) Legislators, senior officials and managers; c) Professionals; d) Technicians and associate professionals; e) Clerks; f) Service works and shop and market sales workers; g) Skilled agricultural and fisher workers; h) Craft and related trade workers; i)Plant and machine operators and assemblers; j) Elementary occupations | Derived from questions on usual occupation or job, description of job and industry they worked in. |

**Table S5:** Predictors tested in the lasso regression

| Ethnicity |
| --- |
| Sex |
| Age |
| **Anthropometry** |
| Height |
| Weight |
| Body mass index |
| Demispan |
| Ulna |
| **Smoking** |
| Ever smoked, cigarettes |
| Ever smoked, pack-years |
| Passive smoke exposure, people smoke where you live |
| Passive tobacco smoke exposure, at home |
| Passive tobacco smoke exposure, in social settings |
| Passive tobacco smoke exposure, elsewhere |
| Paternal smoking, before pregnancy |
| Maternal smoking, before pregnancy |
| Paternal smoking, during pregnancy |
| Paternal smoking, during childhood |
| Maternal smoking, during childhood |
| Paternal smoking, nowadays |
| **Early life influence** |
| Birth weight |
| **Parental immigration status** |
| Both parents born in the United Kingdom |
| One parent born in the United Kingdom |
| No parent born in the United Kingdom |

**Table S6:** Variables with missing data

|  | **White  (N=68)** | **South Asian British (N=22)** |
| --- | --- | --- |
|  | **N (%)** | **N (%)** |
| **Smoking** |  | |
| Father smoking, during pregnancy | 1 (1.5) | 0 (0.0) |
| **(Grand)Parental upbringing** |  | |
| Maternal grandmother | 9 (13.2) | 3 (13.6) |
| Maternal grandfather | 16 (23.5) | 3 (13.6) |
| Paternal grandmother | 12 (17.6) | 2 (9.1) |
| Paternal grandfather | 16 (23.5) | 2 (9.1) |
| **(Grand)Parental educational attainment** |  | |
| Maternal grandmother | 12 (17.6) | 7 (31.8) |
| Maternal grandfather | 15 (22.1) | 5 (22.7) |
| Paternal grandmother | 15 (22.1) | 8 (36.4) |
| Paternal grandfather | 18 (26.5) | 5 (22.7) |
| **Occupation** |  |  |
| Mother | 1 (1.5) | 0 (0.00) |
| Maternal grandmother | 6 (8.8) | 0 (0.00) |
| Maternal grandfather | 8 (11.8) | 4 (18.2) |
| Paternal grandmother | 11 (16.2) | 0 (0.00) |
| Paternal grandfather | 18 (26.5) | 3 (13.6) |

**Table A.7:** Exposure to parental smoking across the life course

|  | **White**  **(N=68)** | **South Asian British (N=22)** |  |
| --- | --- | --- | --- |
|  | **N (%)** | **N (%)** | **P-value** |
| Paternal smoking, before pregnancy | 18 (26.5) | 4 (18.2) | 0.57 |
| Maternal smoking, before pregnancy | 11 (16.2) | 1 (4.5) | 0.28 |
| Paternal smoking, during pregnancy | 7 (10.3) | 0 (0.00) | 0.39 |
| Maternal smoking, during pregnancy | 1 (1.5) | 0 (0.00) | 1.00 |
| Paternal smoking, during childhood* | 7 (10.3) | 2 (9.1) | 1.00 |
| Maternal smoking, during childhood | 4 (5.9) | 1 (4.5) | 1.00 |
| Paternal smoking, nowadays | 5 (7.4) | 1 (4.5) | 1.00 |
| Maternal smoking, nowadays | 2 (2.9) | 1 (4.5) | 1.00 |

*Smoking during pregnancy of one father of a White participant was unknown

**Table S8:** Overview of maternal and maternal grandparents’ occupational status

|  | **Mother** | | **Maternal grandmother** | | **Maternal grandfather** | |
| --- | --- | --- | --- | --- | --- | --- |
|  | **White (N=67)** | **South Asian British (N=22)** | **White (N=62)** | **South Asian British (N=22)** | **White (N=60)** | **South Asian British (N=18)** |
| **ISCO-88 Major Groups** | **N (%)** | **N (%)** | **N (%)** | **N (%)** | **N (%)** | **N (%)** |
| Armed forces | 0 (0.0) | 0 (0.0) | 0 (0.0) | 0 (0.0) | 3 (4.4) | 0 (0.0) |
| Legislators, senior officials and managers | 11 (16.2) | 3 (13.6) | 9 (13.2) | 0 (0.0) | 17 (25.0) | 6 (27.3) |
| Professionals | 35 (51.5) | 8 (36.4) | 16 (23.5) | 3 (13.6) | 17 (25.0) | 6 (27.3) |
| Technicians and associate professionals | 8 (11.8) | 2 (9.1) | 1 (1.5) | 1 (4.5) | 2 (2.9) | 0 (0.0) |
| Clerks | 6 (8.8) | 6 (27.3) | 14 (20.6) | 0 (0.0) | 3 (4.4) | 1 (4.5) |
| Service workers and shop and market sales workers | 3 (4.4) | 1 (4.5) | 3 (4.4) | 0 (0.0) | 1 (1.5) | 0 (0.0) |
| Skilled agricultural and fishery workers | 1 (1.5) | 0 (0.0) | 2 (2.9) | 1 (4.5) | 4 (5.9) | 0 (0.0) |
| Craft and related trade workers | 0 (00) | 0 (0.0) | 1 (1.5) | 1 (4.5) | 4 (5.9) | 1 (4.5) |
| Plant and machine operators and assemblers | 0 (00) | 0 (0.0) | 0 (0.0) | 0 (0.0) | 5 (7.4) | 2 (9.1) |
| Elementary occupations | 1 (1.5) | 0 (0.0) | 1 (1.5) | 1 (4.5) | 4 (5.9) | 2 (9.1) |
| Did not work | 2 (2.9) | 2 (9.1) | 15 (22.1) | 15 (72.0) | 0 (0.0) | 0 (0.0) |
| **P-value** | 0.26 | | 0.001 | | 0.58 | |

**Table S9:** Overview of paternal and paternal grandparents’ occupational status

|  | **Father** | | **Paternal grandmother** | | **Paternal grandfather** | |
| --- | --- | --- | --- | --- | --- | --- |
|  | **White (N=68)** | **South Asian British (N=22)** | **White (N=57)** | **South Asian British (N=22)** | **White (N=50)** | **South Asian British (N=19)** |
| **ISCO-88 Major Groups** | **N (%)** | **N (%)** | **N (%)** | **N (%)** | **N (%)** | **N (%)** |
| Armed forces | 1 (1.5) | 0 (0.0) | 1 (1.5) | 0 (0.0) | 2 (2.9) | 0 (0.0) |
| Legislators, senior officials and managers | 33 (48.5) | 7 (28.0) | 5 (7.4) | 2 (9.1) | 12 (17.6) | 6 (27.3) |
| Professionals | 23 (33.8) | 10 (48.0) | 13 (19.1) | 1 (4.5) | 13 (19.1) | 3 (13.6) |
| Technicians and associate professionals | 6 (8.8) | 1 (4.0) | 4 (5.9) | 0 (0.0) | 2 (2.9) | 1 (4.5) |
| Clerks | 1 (1.5) | 1 (4.0) | 12 (17.6) | 1 (4.5) | 4 (5.9) | 2 (9.1) |
| Service workers and shop and market sales workers | 2 (2.9) | 0 (0.0) | 2 (2.9) | 0 (0.0) | 5 (7.4) | 1 (4.5) |
| Skilled agricultural and fishery workers | 1 (1.5) | 0 (0.0) | 0 (0.0) | 1 (4.5) | 2 (2.9) | 3 (13.6) |
| Craft and related trade workers | 1 (1.5) | 0 (0.0) | 0 (0.0) | 0 (0.0) | 8 (11.8) | 0 (0.0) |
| Plant and machine operators and assemblers | 0 (0.0) | 3 (16.0) | 0 (0.0) | 0 (0.0) | 1 (1.5) | 2 (9.1) |
| Elementary occupations | 0 (0.0) | 0 (0.0) | 2 (2.9) | 1 (4.5) | 1 (1.5) | 1 (4.5) |
| Did not work | 0 (0.0) | 0 (0.0) | 20 (29.4) | 16 (72.7) | 0 (0.0) | 0 (0.0) |
| **P-value** | 0.53 | | 0.001 | | 0.42 | |

**Table S10:** Crude and adjusted difference in FEV1 across categories (categorical variables) and per unit (continuous variables).

|  | **FEV_1_ (L)** | | | |
| --- | --- | --- | --- | --- |
|  | **Crude** | | **Adjusted**^1^ | |
|  | **Difference**  **(95% CI)** | **P-value** | **Difference**  **(95% CI)** | **P-value** |
| **Ethnicity**  **(South Asian)** | -0.71  (-1.10;-0.32) | 0.001 | -0.66  (-0.89;-0.42) | <0.001 |
| **Sex (female)** | -1.34  (-1.56;-1.11) | <0.001 | -0.80  (-1.09;-0.51) | <0.001 |
| **Age (yrs)** | -0.09  (-0.23;0.04) | 0.17 | 0.035  (-0.033;0.10) | 0.31 |
| **Height (cm)** | 0.07  (0.06;0.08) | <0.001 | 0.021  (-0.005;0.048) | 0.11 |
| **Demispan (cm)** | 0.13  (0.10;0.16) | <0.001 | -0.028  (-0.019;0.074) | 0.24 |
| **Father occupational status (≥professional category)** | 0.055  (-0.42; 0.53) | 0.82 | -0.21  (-0.44;-0.023) | 0.08 |
| **Birth weight (kg)** | 0.20  (-0.13;0.54) | 0.15 | -0.005  (-0.18;0.17) | 0.96 |
| **Maternal educational attainment (>High school)** | 0.53  (0.10;0.96) | 0.016 | 0.31  (0.080;0.54) | 0.009 |
| **Maternal upbringing (urban)** | 0.15  (-0.26;0.56) | 0.48 | -0.029  (-0.23;0.17) | 0.77 |

^1^Difference for each predictor adjusted for all other predictors reported in the crude analysis

**Table S11:** Lung function reversibility expressed as percent difference^1^

|  | **Total population** | | | | **White** | | | | **South Asian British** | | | |
| --- | --- | --- | --- | --- | --- | --- | --- | --- | --- | --- | --- | --- |
|  | **N** | **Mean (SD)** | **Min** | **Max** | **N** | **Mean (SD)** | **Min** | **Max** | **N** | **Mean (SD)** | **Min** | **Max** |
| **FVC** | 87.0 | -0.4 (2.6) | -8.7 | 7.3 | 65.0 | -0.6 (2.6) | -8.7 | 7.3 | 22.0 | -0.1 (2.6) | -4.0 | 6.0 |
| **FEV_1_** | 88.0 | -2.6 (4.1) | -9.1 | 23.3 | 67.0 | -3.0 (2.7) | -9.1 | 3.2 | 21.0 | -1.6 (6.9) | -8.2 | 23.3 |
| **FEV_1_/FVC** | 85.0 | -2.1 (3.2) | -7.4 | 19.7 | 64.0 | -2.4 (1.9) | -7.4 | 3.1 | 21.0 | -1.4 (5.5) | -7.1 | 19.7 |

^1^% difference = (pre-bronchodilator – post-bronchodilator)/ post-bronchodilator *100
