# Supplementary material for: Explaining ethnic disparities in lung function among young adults: A pilot investigation
Source: PLoS One. 2017 Jun 2;12(6):e0178962. doi: 10.1371/journal.pone.0178962 (PMC5456386; doi:10.1371/journal.pone.0178962)
Supplement: S2 Text — (PDF) [file pone.0178962.s007.pdf]

ELIGIBILITY QUESTIONNAIRE

(STAGE 1)

Instructions

The inclusion questionnaire will be designed using Qualtrics, which is an online survey system offered by Imperial College London. The inclusion questionnaire will be completed online by the participants and will be available to the participants via the website.

If participants answer ***Yes*** *to Questions 1.1 and 1.2, if they are between 18 and 23 years of age and if they consider themselves as White or South Asian ethnicity (i.e. English/Welsh/Scottish/Northern Irish/British, Irish, Gypsy or Irish Traveller, Any other White background, White and Asian, Indian, Pakistani, Bangladeshi, Any other Asian background) and answer* ***Yes*** *to Question 1.5 then they are eligible to participate in the study and the safety questions will be asked (Questions 2.1 to 2.7). If participants are eligible and it is safe for them to participate then the contact details (Questions 3.1 to 3.5) will be asked. Eligible participants will be informed that they will be contacted by email or phone to book a study visit.*

No safety questions will be asked or contact details will be collected from the participants if they are not eligible to participate. The obtained information would not allow us to track the non-eligible participant and the answers to the questions do not contain any sensitive information.

1. Demographic information

1.1.Are you a student at Imperial College London?

Yes ❑
No ❑

1.2.Are you born in the United Kingdom (England, Scotland, Wales, Northern Ireland)?

Yes ❑
No ❑

1.3. What is your age range?

<18yrs ❑
18-23 ❑
24-27 ❑
>27yrs ❑

1.4. To which of the ethnic group do you consider that you belong?
(Choose one option that best describes your ethnic group or background)

White

English / Welsh / Scottish / Northern Irish / British ❑
Irish ❑
Gypsy or Irish Traveller ❑
Any other White background, please describe: ❑

____

Mixed / Multiple ethnic groups

White and Black Caribbean ❑
White and Black African ❑
White and Asian ❑
Any other Mixed / Multiple ethnic background, please describe: ❑

____

Asian / Asian British

Indian ❑
Pakistani ❑
Bangladeshi ❑
Chinese ❑
Any other Asian background, please describe: ❑

____

Black / African / Caribbean / Black British

Caribbean ❑
African ❑
Any other Black / African / Caribbean background, please describe: ❑

____

Other ethnic group

Arab ❑
Any other ethnic group, please describe: ❑

____

1.5.Were at least three of your grandparents born in United Kingdom, Western-Europe, South-Asia (Pakistan, India, Nepal, Bangladesh, Bhutan, Sri Lanka) or East-Africa (Kenya, Tanzania, Uganda)?

Yes ❑
No ❑

2. Safety Questions

2.1.In the past three months have you had any surgery on your chest or abdomen? Yes ❑ No ❑

2.2. Have you had a heart attack within the past three months? Yes❑ No ❑

2.3.Do you have a detached retina or have you had eye surgery within the past three months? Yes ❑ No ❑

2.4.Have you been hospitalized for any other heart problem within the past month? Yes ❑ No ❑

2.5.Are you in the last trimester of pregnancy? Yes ❑ No ❑

2.6.Are you currently taking medication for tuberculosis? Yes ❑ No ❑

2.7.Have you had a pneumothorax within the past three months? Yes ❑ No ❑

3. Contact details

3.1. What is your Surname? ________________

3.2. What is your Name? ________________

3.3. What is your date of birth?

_____ _____/_____ _____/_____ _____ _____ _____
d d m m y y y y

3.4. What is your Phone number? ________________

3.5. What is your Imperial College London email address? ________________

3.6. When would it suit you to have the study visit?

	Morning (8-11am)	Lunch time (11am-2pm)	Afternoon (2-5pm)
Monday	❑	❑	❑
Tuesday	❑	❑	❑
Wednesday	❑	❑	❑
Thursday	❑	❑	❑
Friday	❑	❑	❑

ID ______ ______ ______ ______ ______ ______

Date:____ ____/____ ____/_____ _____ _____ _____
d d m m y y y y

ELIGIBILITY QUESTIONAIRE
(STAGE 2)

Instructions
The Eligibility questionnaire (stage 2) contains questions which are necessary to assess whether it is safe for the participant to perform spirometry. If any of the Questions 1.1 through 1.9 is ***Yes*** *then it is not safe for the participant to perform the lung function testing. Therefore, the participant will be excluded from the study.*

Safety Questions

1. I just want to check the safety questions that you answered when you volunteered and check that nothing has changed.

1.1. Have you had any surgery on your chest or abdomen?

Yes ❑
No ❑

1.2. Have you had a heart attack within the past three months?

Yes ❑
No ❑

1.3. Do you have a detached retina or have you had eye surgery within the past three months?

Yes ❑
No ❑

1.4. Have you been hospitalized for any other heart problem within the past month?

Yes ❑
No ❑

1.5. Are you in the last trimester of pregnancy?

Yes ❑
No ❑

1.6. Does the participant have a resting pulse of greater than 120 beats per minute?

Yes ❑
No ❑

1.7. Are you currently taking medication for tuberculosis?

Yes ❑
No ❑

1.8. Have you had a pneumothorax within the past three months?

Yes ❑
No ❑

1.9. *Is there some other reason why this participant should not perform the spirometry maneuver?*
If ***yes,*** record reason.

Yes ❑
No ❑

____

____

If the answer to any of Questions 1.2. through 1.9. is "Yes", then the participant will be excluded from the study.

2. Researcher ID ____ ____ ___

ID _____ _____ _____ _____ _____ _____

Date:_____ _____/_____ _____/_____ _____ _____ _____
d d m m y y y y

MEASUREMENT QUESTIONNAIRE

Instructions
The Measurements questionnaire contains questions related to respiratory disease and/or symptoms and will record the results of the anthropometric and cardiovascular measurement and lung function testing.

1. Demography

1.1. What is the participant's sex?

Male ❑
Female ❑

2. Respiratory infection

2.1 Have you had a respiratory infection (cold) in the last three weeks? Yes ❑
No ❑

3. Medications for breathing

3.1. Have you taken any medications for breathing in the last 24 hours? Yes ❑
No ❑

*If **yes**, record name/type of medication(s used.*

____

____

If Question 3.1 is yes and the medication used includes any of the types below, go to question 3.2. If no, go to question 4

TYPE OF MEDICATION	EXAMPLES	
Short-acting beta-2 agonist	albuterol, salbutamol	6 hours prior to clinic visit
Anticholinergic inhaler	Atrovent, ipratropium	6 hours prior to clinic visit
Long-acting beta-2 agonist (including combination preparations that contain a LABA)	Serevent, Advair, formoterol, Symbicort	12 hours prior to clinic visit
Oral beta-2 agonist	Albuterol	12 hours prior to clinic visit
Oral theophylline	Theodur	12-24 hours prior to clinic visit, depending upon preparation
Long-acting anticholinergic	Spiriva, tiotropium	24 hours prior to clinic visit

3.2. Did you use a short acting beta agonist or anticholinergic inhaler, either alone or in combination with some other product, in the last six hours? Yes ❑
No ❑

3.3 Did you use a long acting beta agonist or oral beta 2 agonist, either alone or in combination with some other product, in the last 12 hours? Yes ❑
No ❑

3.4. Did you use an oral theophyllin/ long acting anticholinergic, either alone or in combination with some other product, in the last 24 hours? Yes ❑
No ❑

4. Smoking

4.1. When did you last smoke?
i) ____ ____ ____hrs ago
ii) ____ ____days ago

Enter 999 if non smoker or ex smoker (has not smoked in the last month)

ID ______ ______ ______ ______ ______ ______

Date:______ ______/______ ______/______ ______ ______ ______
d d m m y y y y

5. Cardiovascular measurements

5.1.A. Pulse	___ ____ ____ **bpm**
5.1.B. Pulse	___ ____ ____ **bpm**
5.1.C. Pulse	___ ____ ____ **bpm**
5.2.A. Blood pressure (Syst/Dia)	___ ___ ___/___ ___ __ **mm Hg**
5.2.B. Blood pressure (Syst/Dia)	___ ___ ___/___ ___ __ **mm Hg**
5.2.C. Blood pressure (Syst/Dia)	___ ___ ___/___ ___ __ **mm Hg**

6. Anthropometric measurements

6.1. Height	____ ____ ____**cm**
6.2. Weight	____ ____ ____.____**kg**
6.3.A Long bone length (ulna)	____ ____ ____**cm**
6.3.B Long bone length (ulna)	____ ____ ____**cm**
6.4.A Long bone length (demispan)	____ ____ ____**cm**
6.4.B Long bone length (demispan)	____ ____ ____**cm**

7. Measurements Compliance – Prebronchodilator Spirometry

7.1. Acceptable pre-bronchodilator test completed?

Yes ❑
No ❑

7.2. Unable to obtain satisfactory spirometry (check one)?

The participant did not understand the instructions ❑
The participant was unable to physically cooperate ❑
refused ❑
not applicable ❑

7.3. Were any adverse events related to the spirometry maneuver observed by the evaluator?

Yes ❑
No ❑

If yes, please describe the event

____

____

ID _____ _____ _____ _____ _____ _____

Date:_____ _____/_____ _____/_____ _____ _____ _____
d d m m y y y y

7.4. If the participant had a condition that would affect the result of their spirometry test (e.g., kyphosis, missing limbs, etc) note that condition here

____

____

8. Measurement Compliance - Bronchodilator

8.1. Bronchodilator administered?

Yes ❑
No ❑

9. Measurements Compliance – Postbronchodilator Spirometry

9.1. Acceptable post-bronchodilator test completed?

Yes ❑
No ❑

9.2. Unable to obtain satisfactory spirometry (check one)?

The participant did not understand the instructions ❑
The participant was unable to physically cooperate ❑
refused ❑
not applicable ❑

9.3. Were any adverse events related to the spirometry maneuver observed by the evaluator?

Yes ❑
No ❑

If yes, please describe the event

____

____

9.4. If the participant had a condition that would affect the result of their spirometry test (e.g., kyphosis, missing limbs, etc) note that condition here

____

____

10. Cardiovascular measurements before completing study visit

10.1.A. Pulse ___ ____ ____ **bpm**

10.1.B. Pulse ___ ____ ____ **bpm**

10.2.A. Blood pressure (Syst/Dia) ___ ___ ___/___ ___ __ **mm Hg**

10.2.B. Blood pressure (Syst/Dia) ___ ___ ___/___ ___ __ **mm Hg**

11. Researcher ID

____ ____ ____ ____

ID ______ ______ ______ ______ ______ ______
Date:______ ______/______ ______/______ ______ ______ ______
d d m m y y y y

CORE QUESTIONNAIRE

PART A – Respiratory Symptoms, disorders and comorbidities
These questions pertain mainly to your chest. Please answer yes or no if possible. If you are in doubt about whether your answer is yes or no, please answer no.

Cough

1. Do you usually cough when you don't have a cold? — Yes ☐ No ☐
*[If **yes**, continue with Question 1A; If **no**, skip to Question 2]*

1A. Are there months in which you cough on most days? — Yes ☐ No ☐
*[If **yes**, ask both Questions 1B & 1C; If **no**, skip to Question 1]*

1B. Do you cough on most days for as much as three months each year? — Yes ☐ No ☐

1C. For how many years have you had this cough? — Less than 2 years ☐ 2-5 years ☐ More than 5 years ☐

Phlegm

2. Do you usually bring up phlegm from your chest, or do you usually have phlegm in your chest that is difficult to bring up when you don't have a cold? — Yes ☐ No ☐
*[If **yes**, continue with Question 2A; If **no**, skip to Question 3]*

2A. Are there months in which you have this phlegm on most days? — Yes ☐ No ☐
*[If **yes**, ask both Questions 2B & 2C; If **no**, skip to Question 3]*

2B. Do you bring up this phlegm on most days for as much as three months each year? — Yes ☐ No ☐

2C. For how many years have you had this phlegm? — Less than 2 years ☐ 2-5 years ☐ More than 5 years ☐

Wheezing/Whistling

3. Have you had wheezing or whistling in your chest at any time in the last 12 months? — Yes ☐ No ☐
*[If **yes**, ask both Questions 3A & 3B; If **no**, skip to Question 4]*

3A. In the last 12 months, have you had this wheezing or whistling only when you have a cold? — Yes ☐ No ☐

3B. In the last 12 months, have you ever had an attack of wheezing or whistling that has made you feel short of breath? — Yes ☐ No ☐

Breathlessness

4. Are you unable to walk due to a condition other than shortness of breath? Yes ☐ No ☐
[*If **yes** to Question 4, please describe this condition on the line below and then skip to Question 5. If **no*** or unsure*, go directly to Question 20.]*

Nature of condition(s): ________________________________

5. Are you troubled by shortness of breath when hurrying on the level or walking up a slight hill? Yes ☐ No ☐
*[If **yes**, ask Question 5A through 5D; If **no**, skip to Question 6]*

5A. Do you have to walk slower than people of your age on level ground because of shortness of breath? Yes ☐ No ☐ Does not apply ☐

5B. Do you ever have to stop for breath when walking at your own pace on level ground? Yes ☐ No ☐ Does not apply ☐

5C. Do you ever have to stop for breath after walking about 100 yards (or after a few minutes) on level ground? Yes ☐ No ☐ Does not apply ☐

5D. Are you too short of breath to leave the house or short of breath on dressing or undressing? Yes ☐ No ☐ Does not apply ☐

Diagnosed lung disease

6. Has a doctor or other health care provider ever told you that you have asthma, asthmatic bronchitis or allergic bronchitis? Yes ☐ No ☐
*[If **yes**, ask Question 6A. If **no**, skip to Question 7]*

6A. Do you still have asthma, asthmatic bronchitis or allergic bronchitis? Yes ☐ No ☐

7. Has a doctor or other health care provider ever told you that you have chronic bronchitis? Yes ☐ No ☐
*[If **yes**, ask Question 7A. If **no**, skip to Question 8]*
7A. Do you still have chronic bronchitis? Yes ☐ No ☐

Exacerbations

8. Have you ever had a period when you had breathing problems that got so bad that they interfered with your usual daily activities or caused you to miss work? *[If **yes**, ask Question 8A. If **no**, skip to Question 9]* Yes ☐ No ☐

8A. How many such episodes have you had in the past 12 months?
[If 25A >0, ask Questions 8B and 8C, else skip to Question 9] ____ ____ episodes

8B. For how many of these episodes did you need to see a doctor or other health care provider in the past 12 months? ____ ____ episodes

8C. For how many of these episodes were you hospitalized overnight in the past 12 months?
[If 8C >0, ask Question 8C1, else skip to Question 9] _____ _____ episodes

8C1. All together, for how many total days were you hospitalized overnight for breathing problems in the past 12 months? ____ ____ ____ days

Comorbidities

9. Has a doctor or other health care provide ever told you that you had:

	Yes	No
A. Hypertension	☐	☐
B. Diabetes	☐	☐
C. Tuberculosis	☐	☐

*[If **yes** to 9C, then ask 9C1; otherwise, skip to Question 10]*

9C1. Are you currently taking medicine for tuberculosis? Yes ☐
*[If **no** to 9C1, then ask 9C2; otherwise, skip to Question 10]* No ☐

9C2. Have you ever taken medicine for tuberculosis? Yes ☐
No ☐

10. Have you ever had an operation on your chest in which a part of your lung was removed?
Yes ☐
No ☐

11. Were you hospitalized as a child for breathing problems prior to the age of 10?
Yes ☐
No ☐
Don't Know ☐

12. Has a doctor or other health care professional told your father, mother, sister or brother that they had a diagnosis of emphysema, chronic bronchitis or COPD? Yes ☐
No ☐

ID ____ ____ ____ ____ ____ ____
Date: ____ ____/____ ____/____ ____ ____ ____
d d m m y y y y

PART B - Quality of life

13. In general, would you say your health is: *(Check one)*

Excellent ☐
Very good ☐
Good ☐
Fair ☐
Poor ☐

14. The following questions are about activities you might do during a typical day. Does ***your health now limit*** you in these activities? If so, how much?

	Yes, limited a lot	Yes, limited a little	Not limited at all
14a. ***Moderate activities,*** such as moving a table, pushing a vacuum cleaner, bowling or playing golf	☐	☐	☐
14b.Climbing **several** flights of stairs	☐	☐	☐

15. **During *the past 4 weeks*,** have you had any of the following problems with your work or other regular daily activities **as a result of your physical health**?

	No, none of the time	Yes, a little of the time	Yes, some of the time	Yes, most of the time	Yes, all the time
15a. Accomplished **less** than you would like	☐	☐	☐	☐	☐
15b. Were limited in the **kind** of work or other activities	☐	☐	☐	☐	☐

16. **During *the past 4 weeks***, have you had any of the following problems with your work or other regular daily activities **as a result of any emotional problems** (such as feeling depressed or anxious)?

	No, none of the time	Yes, a little of the time	Yes, some of the time	Yes, most of the time	Yes, all the time
16a. **Accomplished less** than you would like	☐	☐	☐	☐	☐
16b. Didn't do work or other activities as **carefully** as usually	☐	☐	☐	☐	☐

17. **During the *past 4 weeks*,** how much did **pain** interfere with your normal work (including both work outside the home and housework)?

Not at all ☐
Slightly ☐
Moderately ☐
Quite a bit ☐
Extremely ☐

ID ______ ______ ______ ______ ______ ______
Date:______ ______/______ ______/______ ______ ______ ______
d d m m y y y y

18. These questions are about how you feel and how things have been with you **during the past 4 weeks**. For each question, please give the one answer that comes closest to the way you have been feeling. How much of the time during the **past 4 weeks**

	All of the time	Most of the time	A good bit of the time	Some of the time	A little of the time	None of the time
18a.Have you felt calm and peaceful?	☐	☐	☐	☐	☐	☐
18b.Did you have a lot of energy?	☐	☐	☐	☐	☐	☐
18c.Have you felt downhearted and blue?	☐	☐	☐	☐	☐	☐

19. **During the *past 4 weeks***, how much of the time has your **physical health or emotional problems** interfered with your social activities (like visiting with friends, relatives, etc.)?

All the time ☐
Most of the time ☐
Some of the time ☐
A little of the time ☐
None of the time ☐

20. Now we'd like to ask you some questions about how your health may have changed. Compared to one year ago,

	Much better	Slightly better	About the same	Slightly worse	Much worse
20a.How would you rate your **physical health** in general now?	☐	☐	☐	☐	☐
20b.How would you rate your **emotional problems** (such as feeling anxious, depressed or irritable) **now** ?	☐	☐	☐	☐	☐

ID ____ ____ ____ ____ ____ ____

Date:____ ____/____ ____/____ ____ ____ ____
d d m m y y y y

PART C - Management Section

Now I am going to ask you about medicines that you may be taking to help with your breathing. I want to know about medicines that you take on a regular basis and medicines that you may take only for the relief of symptoms. I would like you to tell me each medicine that you take, what form do you take it in, and how often you take it each month.

21. In the past 12 months, have you taken any medications for your breathing (including medications for nasal congestion)? Yes ☐
If participant does not take any medications to help their breathing, skip to Question 27. No ☐

21.A. Medication Name (not entered)				
21.B. Medication Code	__ __ __	__ __ __	__ __ __	__ __ __
21.C. Formulation	Pills ☐<br>Inhaler ☐<br>Nebulizer ☐<br>Liquid ☐<br>Suppository ☐<br>Injection ☐<br>Other ☐	Pills ☐<br>Inhaler ☐<br>Nebulizer ☐<br>Liquid ☐<br>Suppository ☐<br>Injection ☐<br>Other ☐	Pills ☐<br>Inhaler ☐<br>Nebulizer ☐<br>Liquid ☐<br>Suppository ☐<br>Injection ☐<br>Other ☐	Pills ☐<br>Inhaler ☐<br>Nebulizer ☐<br>Liquid ☐<br>Suppository ☐<br>Injection ☐<br>Other ☐
21.D. Is the medicine taken on most days or just when you have symptoms or both? *(If 'most day ask Q26E if both ask 'both' 26E and Q26F)*	Most days ☐<br>Symptoms ☐<br>Both ☐<br>Other ☐	Most days ☐<br>Symptoms ☐<br>Both ☐<br>Other ☐	Most days ☐<br>Symptoms ☐<br>Both ☐<br>Other ☐	Most days ☐<br>Symptoms ☐<br>Both ☐<br>Other ☐
21.E. When you are taking the medication how many days a week do you take it?	__ days/week	__ days/week	__ days/week	__ days/week
21.F. When you are taking the medication, how many months in the past 12 months have you taken it?	0-3 ☐<br>4-6 ☐<br>7-9 ☐<br>10-12 ☐	0-3 ☐<br>4-6 ☐<br>7-9 ☐<br>10-12 ☐	0-3 ☐<br>4-6 ☐<br>7-9 ☐<br>10-12 ☐	0-3 ☐<br>4-6 ☐<br>7-9 ☐<br>10-12 ☐

22. Please tell me about any other products that you take or things you do to help your breathing that you have not already told me about.

**Medicine or Activity**	**Code**
	____ ____ ____
	____ ____ ____
	____ ____ ____
	____ ____ ____

ID ______ ______ ______ ______ ______ ______
Date:______ ______/______ ______/______ ______ ______ ______
d d m m y y y y

PART D - Smoking

Now I am going to ask you about smoking. First I will ask about cigarettes, including hand rolled cigarettes, and then I will ask about other items that are smoked.

23.1. Have you ever smoked **cigarettes**? Yes ☐ No ☐

("Yes," means more than 20 packs of cigarettes in a lifetime or more than 1 cigarette each day for a year)
*[if **yes**, ask questions 23.2 through 23.5; otherwise, skip to Question 24.1)*

23.2. How old were you when you first started regular cigarette smoking? ____ _____ years old

23.3. If you have stopped smoking, how old were you when you last stopped? _________ years old
(If the participant has not stopped smoking, record as code '999'.)

23.4. On average over the entire time that you smoke(d), about how many cigarettes per day/per week do (did) you smoke? i)____ ____ ____ cigarettes/day
ii)___ ____ ____ cigarettes/week

23.5. On average over the entire time that you smoke(d), do (did) you primarily smoke manufactured or hand-rolled cigarettes? Manufactured ☐ Hand-rolled ☐

24.1. Have you ever smoked a **water pipe**? Yes ☐ No ☐

("Yes," means more than 1 water pipe per week for one year at any time in your life)
*[if **yes**, ask questions 24.2 through 24.4; otherwise, skip to Question 25.1)*

24.2. How old were you when you first started regular water pipe smoking? ____ _____ years old

24.3. If you have stopped smoking, how old were you when you last stopped? _____ ____ years old
(If the participant has not stopped smoking, record as code '999'.)

24.4. On average over the entire time that you smoke(d), about how many water pipe per day/per week do (did) you smoke? i)___ ____ ____ water pipes /day
ii)___ ____ ____water pipes /week

Second hand smoke exposure

25.1. Not counting yourself, how many people in your household smoke regularly? ___ ___

25.2. Do people smoke regularly in the room where you work? Yes ☐ No ☐ Don't work ☐

25.3. How many hours per day, are you exposed to other people's tobacco smoke in the following locations?
- 25.3.1. At home __ __hours
- 25.3.2. In workplace __ __hours
- 25.3.3. Bars, restaurants, cinemas or similar social settings __ __hours
- 25.3.4. Elsewhere __ __hours

ID ______ ______ ______ ______ ______ ______

Date:______ ______/______ ______/______ ______ ______ ______
d d m m y y y y

PART E – Family history

Family Composition

26. How many siblings do you have? __________

If the answer to Question 26 is >0 then ask Question 27 to 28, otherwise skip to Question 29.1.

27. How many older siblings do you have? ____________

28. How many older brothers do you have? ____________

Family history – Education

29.1 What is the highest level of schooling your mother has completed?

- Primary School ☐
- Middle School ☐
- High School ☐
- Some College (Trade/Professional/Community) ☐
- Four Year College/University ☐
- None ☐
- Unknown ☐

29.2 What is the highest level of schooling your maternal grandmother has completed?

- Primary School ☐
- Middle School ☐
- High School ☐
- Some College (Trade/Professional/Community) ☐
- Four Year College/University ☐
- None ☐
- Unknown ☐

29.3 What is the highest level of schooling your maternal grandfather has completed?

- Primary School ☐
- Middle School ☐
- High School ☐
- Some College (Trade/Professional/Community) ☐
- Four Year College/University ☐
- None ☐
- Unknown ☐

29.4 What is the highest level of schooling your father has completed?

- Primary School ☐
- Middle School ☐
- High School ☐
- Some College (Trade/Professional/Community) ☐
- Four Year College/University ☐
- None ☐
- Unknown ☐

29.5 What is the highest level of schooling your paternal grandmother has completed?

- Primary School ☐
- Middle School ☐
- High School ☐
- Some College (Trade/Professional/Community) ☐
- Four Year College/University ☐
- None ☐
- Unknown ☐

29.6 What is the highest level of schooling your paternal grandfather has completed?

- Primary School ☐
- Middle School ☐
- High School ☐
- Some College (Trade/Professional/Community) ☐
- Four Year College/University ☐
- None ☐
- Unknown ☐

Family history – Occupation

30.1. What has been your father's usual occupation or job? ______________
If the he has never worked then enter "never worked" and go to question 31.1

30.2. What does/did he actually do in his work (describe) ________________________
____

30.3. What industry does he work in? ______________
If father unemployed put 999

30.4. For how many years has your father worked at this job? _______ _______ years

30.5. Is your father:

- A manager working for an employer? ☐
- A foreman or supervisor working for an employer? ☐
- Working for an employer, but neither a manager, supervisor or foreman? ☐
- Self-employed? ☐

30.6. Enter ISCO code here: __ __ __ __

31.1. What has been your paternal grandfather's usual occupation or job?______________
If the he has never worked then enter "never worked" and go to question 32.1

31.2. What does/did he actually do in his work (describe) ________________________
____

31.3. What industry did he work in? ______________
If grandfather unemployed put 999

31.4. For how many years has your paternal grandfather worked at this job?_______ _______ years

31.5. Has your paternal grandfather been a farmer?
If yes go to Question 31.6., otherwise skip to Question 31.7.

- Yes ☐
- No ☐

31.6. Has your paternal grandfather been:
If Question 31.6. is being completed then skip to 32.1.

- An owner, but didn't work on the farm ☐
- An owner, but worked on the farm ☐
- A tenant, worked on the farmer but didn't own the farm ☐
- Employed on the farm but not a tenant or owner ☐

31.7. Was your paternal grandfather:
A manager working for an employer? ☐
A foreman or supervisor working for an employer? ☐
Working for an employer, but neither a manager, supervisor or foreman? ☐
Self-employed? ☐

31.8. Enter ISCO code here: __ __ __ __

32.1. Did your paternal grandmother work outside the home?
Yes ☐
No ☐
*If Question 32.1 is **Yes** then go to Question 32.2 to 32.9, otherwise skip to Question 33.1*

32.2. What was your paternal grandmother's usual occupation or job? _______________

32.3. What does/did she actually do in her work (describe) __________________________
____

32.4. What industry did she work in? _______________
If grandmother unemployed put 999

32.5. For how many years has your paternal grandmother worked at this job?________ ________ years

32.6. Has your paternal grandmother been a farmer?
If yes go to 32.7. else skip to 32.8. Yes ☐
No ☐
32.7. Has your paternal grandmother been:
If Question 32.7. is being completed then skip to 33.1.
An owner, but didn't work on the farm ☐
An owner, but worked on the farm ☐
A tenant, worked on the farmer but didn't own the farm ☐
Employed on the farm but not a tenant or owner ☐
32.8. Was your paternal grandmother:
A manager working for an employer? ☐
A foreman or supervisor working for an employer? ☐
Working for an employer, but neither a manager, supervisor or foreman? ☐
Self-employed? ☐
32.9. Enter ISCO code here: __ __ __ __

33.1. Has or does your mother work outside the home?
*If Question 33.1. is **Yes** then go to Question 33.2 to 33.7,* Yes ☐
otherwise skip to Question 34.1 No ☐

33.2. What has been your mother's usual occupation or job? _______________

33.3. What does/did she actually do in her work (describe) ___________________________
____

33.4. What industry does she work in? _______________
If mother unemployed put 999

33.5. For how many years has your mother worked at this job? ________ ________ years

33.6. Is your mother:

A manager working for an employer? ☐
A foreman or supervisor working for an employer? ☐
Working for an employer, but neither a manager, supervisor or foreman? ☐
Self-employed? ☐

33.7. Enter ISCO code here: __ __ __ __

34.1. What has been your maternal grandfather's usual occupation or job?_______________
If the he has never worked then enter "never worked" and go to question 35.1

34.2. What does/did he actually do in his work (describe) ___________________________
____

34.3. What industry did he work in? _______________
If grandfather unemployed put 999

34.4. For how many years has your maternal grandfather worked at this job?________ ________ years

34.5. Has your maternal grandfather been a farmer?
If yes go to Question 34.6., otherwise skip to Question 34.7.

Yes ☐
No ☐

34.6. Has your maternal grandfather been:
If Question 34.6. is being completed then skip to 35.1

An owner, but didn't work on the farm ☐
An owner, but worked on the farm ☐
A tenant, worked on the farmer but didn't own the farm ☐
Employed on the farm but not a tenant or owner ☐

34.7. Was your maternal grandfather:

A manager working for an employer? ☐
A foreman or supervisor working for an employer? ☐
Working for an employer, but neither a manager, supervisor or foreman? ☐
Self-employed? ☐

34.8. Enter ISCO code here: __ __ __ __

35.1. Did your maternal grandmother work outside the home?

Yes ☐
No ☐

*If Question 35.1 is **Yes** then go to Question 35.2 to 35.9, otherwise skip to Question 36.1*

35.2. What was your maternal grandmother's usual occupation or job? _______________

35.3. What does/did she actually do in her work (describe) ___________________________
____

35.4. What industry did she work in? _______________
If grandmother unemployed put 999

35.5. For how many years has your maternal grandmother worked at this job?________ ________ years

35.6. Has your maternal grandmother been a farmer?
If yes go to 35.6. else skip to 35.8.
Yes ☐
No ☐

35.7. Has your maternal grandmother been:
If Question 35.7. is being completed then skip to 36.1.
An owner, but didn't work on the farm ☐
An owner, but worked on the farm ☐
A tenant, worked on the farmer but didn't own the farm ☐
Employed on the farm but not a tenant or owner ☐

35.8. Was your maternal grandmother:
A manager working for an employer? ☐
A foreman or supervisor working for an employer? ☐
Working for an employer, but neither a manager, supervisor or foreman? ☐
Self-employed? ☐

35.9. Enter ISCO code here: __ __ __ __

Family history – Place of birth

36.1. Where was your mother born? City/region:_____________ Country:_____________

36.2. Where was your maternal grandmother born? City/region:_____________ Country:_____________

36.3. Where was your maternal grandfather born? City/region:_____________ Country:_____________

36.4. Where was your father born? City/region:_____________ Country:_____________

36.5. Where was your paternal grandmother born? City/region:_____________ Country:_____________

36.6. Where was your paternal grandfather born? City/region:_____________ Country:_____________

Family history – Place of childhood

37.1. What term best describes the place your mother lived most of the time during her childhood?
Farm ☐
Village in rural area ☐
Small town ☐
Suburb or a city ☐
Don't know ☐

37. 2. What term best describes the place your maternal grandmother lived most of the time during her childhood?
Farm ☐
Village in rural area ☐
Small town ☐
Suburb or a city ☐
Don't know ☐

37. 3. What term best describes the place your <u>maternal grandfather</u> lived most of the time during his childhood?

Farm ☐
Village in rural area ☐
Small town ☐
Suburb or a city ☐
Don't know ☐

37. 4. What term best describes the place your <u>father</u> lived most of the time during his childhood?

Farm ☐
Village in rural area ☐
Small town ☐
Suburb or a city ☐
Don't know ☐

37. 5. What term best describes the place your <u>paternal grandmother</u> lived most of the time during her childhood?

Farm ☐
Village in rural area ☐
Small town ☐
Suburb or a city ☐
Don't know ☐

37. 6. What term best describes the place your <u>paternal grandfather</u> lived most of the time during his childhood?

Farm ☐
Village in rural area ☐
Small town ☐
Suburb or a city ☐
Don't know ☐

Family history – Smoking

38.1. Did your <u>mother</u> smoke regularly before your pregnancy?

Yes ☐
No ☐
Don't know ☐

38.2. Did your <u>father</u> smoke regularly before your pregnancy?

Yes ☐
No ☐
Don't know ☐

38.3. Did your <u>mother</u> smoke regularly during your pregnancy?

Yes ☐
No ☐
Don't know ☐

38.4. Did your <u>father</u> smoke regularly during your pregnancy?

Yes ☐
No ☐
Don't know ☐

38.5. Did your <u>mother</u> smoke regularly during your childhood?

Yes ☐
No ☐
Don't know ☐

38.6. Did your <u>father</u> smoke regularly during your childhood?

Yes ☐
No ☐
Don't know ☐

38.7. Does your <u>mother</u> smoke regularly nowadays?

Yes ☐
No ☐
Don't know ☐

38.8. Does your <u>father</u> smoke regularly nowadays?

Yes ☐
No ☐
Don't know ☐

PART F - Birth linkage, GP and withdrawal details

Birth linkage

This section will only be completed if the participant consents to accessing birth weight information (See consent form).

39. Is your birth name different from your current name? Yes ☐ No ☐

*If the answer to Questions 39 is "Yes", then proceed with Questions 39.1.and 39.2. If the answer to Question 39 is **No** then skip to Question 40.*

39.1. What was your Birth Surname? ________________

39.2. What was your Birth Name? ________________

40. What is your NHS number? ___ ___ ___ /___ ___ ___/___ ___ ___ ___

General practice contact details

This section will only be completed if the participant consents to sending the results of the lung function testing to their GP (See consent form).

41.1. What is the name of your GP practice?

____

41.2. What is the contact address of your GP practice?

____

____

____

Withdrawal

42. Define the circumstance under which the participant withdrew if the participant withdrew

____

____

____

53. Researcher ID ____ ____ ____ ____
